# Clinical significance and functional characterization of RRN3 in gastric cancer: insights from pan-cancer analysis and experimental validation

**DOI:** 10.3389/fonc.2026.1832473

**Published:** 2026-05-22

**Authors:** Ruofan He, Qingqing Xing, Songyi Liu, Shiwei Lin, Xiang Lin, Jianxin Ye

**Affiliations:** 1 Department of Gastrointestinal Surgery 2 Section, The First Affiliated Hospital of Fujian Medical University, Fuzhou, China; 2 Key Laboratory of Gastrointestinal Cancer (Fujian Medical University), Ministry of Education, Fuzhou, China; 3 Department of Internal Medicine-Oncology, Fujian Cancer Hospital and Fujian Medical University Cancer Hospital, Fuzhou, China; 4 Department of Gastrointestinal Surgery, National Regional Medical Center, Binhai Campus of the First Affiliated Hospital, Fujian Medical University, Fuzhou, China; 5 Department of Urology, Fuzhou Hospital of Traditional Chinese Medicine Affiliated to Fujian University of Traditional Chinese Medicine, Fuzhou, China; 6 Department of Gastrointestinal Surgical Oncology, Clinical Oncology School of Fujian Medical University, Fujian Cancer Hospital, Fuzhou, China

**Keywords:** gastric cancer, immune infiltration, pan-cancer analysis, prognosis, ribosome biogenesis, RRN3

## Abstract

**Introduction:**

RRN3 is a nucleolar protein required for ribosome biogenesis, but its role in cancer remains insufficiently defined. This study aimed to systematically evaluate the clinical relevance, molecular characteristics, immune-related features, and biological function of RRN3 across cancers, with a particular focus on gastric cancer (GC).

**Methods:**

Public databases were used to analyze RRN3 expression, clinicopathological associations, survival outcomes, ROC curves, genomic alterations, epigenetic and epitranscriptomic features, immune-related characteristics, drug-response profiles, and co-expression networks. Functional enrichment analysis and a putative ceRNA regulatory network were further explored. Clinical tissue validation, in vitro rescue experiments, and in vivo xenograft assays were used to evaluate the involvement of RRN3 in GC progression.

**Results:**

Pan-cancer analysis showed that RRN3 was upregulated in multiple tumor types, including GC, and that high RRN3 expression was associated with unfavorable overall survival in several cancers. In GC, RRN3 expression was associated with RNA methylation regulators, immune checkpoint genes, tumor mutation burden, microsatellite instability, immune cell infiltration, and drug-response-related signatures. Enrichment analyses suggested that RRN3-associated genes were mainly involved in nucleocytoplasmic transport, spliceosome function, mRNA surveillance, and ribosome biogenesis. Clinical validation showed that RRN3 was upregulated in GC tissues and associated with several clinicopathological features related to tumor progression. Functional experiments showed that RRN3 knockdown inhibited GC cell proliferation, colony formation, migration, and invasion, whereas RRN3 re-expression partly reversed these effects. In vivo xenograft experiments further supported the role of RRN3 in promoting tumor growth.

**Discussion:**

These findings suggest that RRN3 is associated with GC progression and may represent a candidate molecule for further investigation in GC.

## Introduction

Gastric cancer (GC) remains one of the leading causes of cancer-related mortality worldwide, particularly imposing a high disease burden in developing countries (1–3). Owing to its insidious onset and marked metastatic potential, most GC cases are diagnosed at advanced stages, which markedly limits the effectiveness of current therapeutic strategies. Although endoscopic and imaging techniques are available for early detection, their sensitivity, specificity, and accessibility remain suboptimal. Therefore, the identification of novel molecular factors associated with GC progression remains important for improving early diagnosis and expanding therapeutic options for GC.

RRN3, also known as TIF-IA, is a critical regulatory factor in ribosomal RNA (rRNA) transcription. As a transcription initiation factor for RNA polymerase I (Pol I), RRN3 recruits Pol I to ribosomal DNA (rDNA) promoter regions to initiate rRNA transcription, thereby playing an essential role in ribosome biogenesis and cell proliferation (4, 5). Dysregulation of the RRN3/Pol I axis has been implicated in several pathological processes, including neurological disorders and cellular stress responses (6–8). Recent studies have also linked abnormal ribosome biogenesis and Pol I transcriptional activity to cancer progression (9). RRN3 may contribute to this process because of its central role in Pol I-dependent rRNA transcription. For example, elevated RRN3 expression has been associated with enhanced proliferation, invasiveness, chemoresistance, and poor prognosis in pancreatic cancer (10). CX-5461, a selective Pol I inhibitor, suppresses RRN3-dependent transcription initiation, leading to reduced rRNA synthesis, nucleolar stress, DNA damage, and cancer cell death (11). In colorectal cancer, pharmacological doses of aspirin have been shown to induce RRN3 degradation, inhibit rDNA transcription, and promote tumor cell apoptosis (12). In addition, RRN3 has been reported to function as a downstream effector of c-Myc-mediated metabolic activation and ribosome biogenesis in medulloblastoma (13). More recently, EGR1 was shown to promote Pol I-directed transcription and cancer cell growth partly by activating RRN3 expression, further supporting the relevance of RRN3-related transcriptional regulation in cancer (14). In addition to its canonical role in rRNA transcription, recent evidence suggests that RRN3 may also participate in stress-induced RNA processing programs. For example, nutrient stress was reported to divert RRN3 from rRNA transcription to alternative polyadenylation regulation of autophagy-related mRNAs in ovarian cancer (15).

With the rapid development of multi-omics datasets and computational biology tools, pan-cancer analysis has become a powerful strategy for investigating gene expression patterns, genetic alterations, clinical relevance, and immune characteristics across different tumor types (16–19). Compared with studies focused on a single cancer type, pan-cancer analyses provide broader insights into shared oncogenic mechanisms and tumor-specific pathways, thereby offering a systematic framework for biomarker discovery and hypothesis generation. Given its conserved role in ribosome biogenesis and the frequent activation of rRNA transcription in highly proliferative tumors, RRN3 represents a biologically relevant candidate for investigation across multiple cancer types.

**Figure 1:**
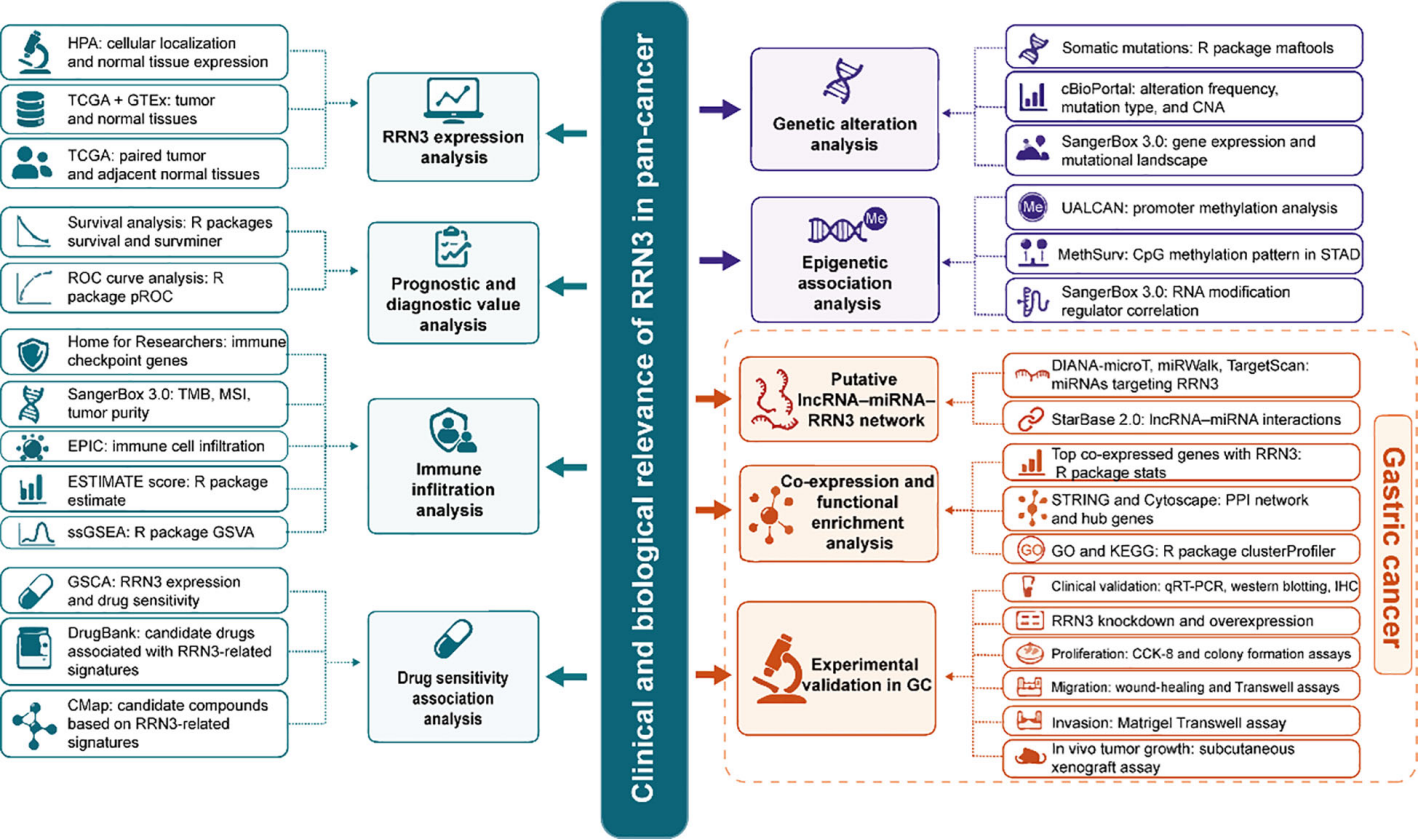
Overview of the study design and analytical workflow. Schematic diagram of the overall study design, including pan-cancer expression profiling, survival and ROC curve analyses, immune infiltration analysis, drug sensitivity association analysis, genetic and epigenetic alteration analyses, construction of a putative ceRNA regulatory network, functional enrichment analysis, and experimental validation in gastric cancer. Clinical tissue validation, *in vitro* rescue experiments, and *in vivo* xenograft assays were performed to further assess the role of RRN3 in gastric cancer progression.

Although previous studies have suggested a potential role for RRN3 in cancer (20–22), its clinical relevance and biological function in GC remain poorly understood, particularly from a pan-cancer perspective. In this study, we systematically analyzed *RRN3* expression across multiple tumor types and evaluated its clinicopathological relevance, survival, and ROC curve profiles. We further investigated genomic alterations, epigenetic features, immune-related characteristics, drug sensitivity associations, and co-expression patterns related to RRN3, and constructed a putative ceRNA regulatory network in GC. In addition, clinical tissue validation, functional enrichment analysis, *in vitro* rescue experiments, and *in vivo* xenograft experiments were performed to further assess the role of RRN3 in GC progression. This study aimed to evaluate the clinical and biological relevance of RRN3 in cancer, with a particular focus on GC. The overall study design is shown in **Figure 1**.

## Materials and methods

### Subcellular localization and expression of RRN3

To investigate the subcellular localization of RRN3 protein, we retrieved immunofluorescence images from the HPA database for HEK293, MCF-7, and U2OS cell lines. We also used the GTEx data integrated in the HPA database to analyze *RRN3* mRNA expression across normal human tissues. For differential expression analysis between normal and tumor tissues, we acquired uniformly processed RNA sequencing data in Transcripts Per Million (TPM) format from TCGA and GTEx cohorts via the UCSC XENA platform (https://xenabrowser.net/datapages/), with preprocessing conducted using the Toil pipeline. Expression values were transformed using the formula log2(TPM + 1). Comparisons between tumor and normal tissues were based on the combined TCGA-GTEx dataset, while matched tumor-adjacent normal tissue comparisons were restricted to paired samples from TCGA. All statistical analyses were conducted in R software utilizing the stats, car, and ggplot2 packages. Group differences were assessed using the Wilcoxon rank-sum test, and results were visualized accordingly. To further investigate the relationship between *RRN3* expression and tumor stage, we utilized the “Stage Plot” module available on the GEPIA2 platform.

### Survival and ROC curve analyses of RRN3

RNA-seq data in TPM format from the TCGA cohort were retrieved and log2-transformed as log2(TPM + 1). The Xiantao platform was used to perform survival and ROC curve analyses of RRN3 across multiple cancer types. For survival analysis, Cox regression models were applied to assess the association between *RRN3* expression and patient survival, and hazard ratios (HRs), 95% confidence intervals (CIs), and P-values were visualized using forest plots. Kaplan–Meier survival curves were generated to compare survival outcomes between the high- and low-*RRN3*-expression groups, and statistical significance was determined using the log-rank test. ROC curves were used to evaluate the ability of RRN3 expression to distinguish tumor tissues from normal tissues, and the area under the curve (AUC) was calculated for each cancer type. An AUC > 0.75 was considered to indicate relatively good tumor-normal separation in the analyzed datasets.

### Genetic alteration analysis of *RRN3*

The genetic alteration landscape of *RRN3* was analyzed using cBioPortal based on data from TCGA and the Pan-Cancer Analysis of Whole Genomes (PCAWG) project of the International Cancer Genome Consortium (ICGC). A total of 2,683 samples from 2,565 patients were included in the analysis. Using the “Cancer Types Summary” module, we examined the mutation types, copy number alterations (CNAs), and alteration frequencies of *RRN3* across the TCGA PanCancer Atlas studies. The “Plots” module was used to evaluate the association between *RRN3* mRNA expression and CNAs. Somatic mutation data for stomach adenocarcinoma (STAD) were further obtained from the TCGA cohort. STAD samples were divided into high- and low-*RRN3*-expression groups according to *RRN3* expression levels. The SangerBox 3.0 platform was then used to explore the associations between *RRN3* expression and mutations in key cancer-related genes. Differences in mutation frequencies between the two groups were assessed using the chi-square test.

### DNA methylation and mRNA modification analysis

The DNA methylation level of the *RRN3* promoter in normal and tumor tissues across multiple cancer types was evaluated using UALCAN based on TCGA methylation data. Methylation levels were quantified using β-values, with values ranging from 0.25 to 0.30 considered indicative of hypomethylation and values ranging from 0.50 to 0.70 considered indicative of hypermethylation. Statistical significance was assessed using the Wilcoxon rank-sum test. For stomach adenocarcinoma (STAD), the “Gene Visualization” module in MethSurv was used to generate detailed plots and heatmaps of methylation levels at *RRN3* CpG sites. This site-specific CpG methylation analysis was performed as an exploratory analysis of methylation patterns within the RRN3 promoter region.

The mRNA Modification Analysis module of the SangerBox 3.0 platform was used to investigate the associations between *RRN3* and 45 RNA methylation regulators across multiple cancer types. This analysis was based on RNA-seq data from the combined TCGA–GTEx dataset after log2(x + 1) normalization. Pearson correlation coefficients were calculated to evaluate the expression correlations between RRN3 and RNA methylation regulators.

### Immune-related correlation analysis of *RRN3*

To investigate the immune-related associations of RRN3, the “Pan-Cancer Immuno-Correlation” module on the Home-for-Researchers platform was used to analyze the correlations between RRN3 expression and eight immune checkpoint genes across TCGA cancer types. The “Pan-Cancer Heterogeneity Analysis” module in SangerBox 3.0 was used to evaluate the associations between RRN3 expression and tumor mutation burden (TMB), microsatellite instability (MSI), and tumor purity across multiple cancers. To further assess the association between RRN3 expression and immune cell infiltration, the “Immune Cell Analysis (EPIC)” tool in SangerBox 3.0 was applied using uniformly processed TCGA-TARGET-GTEx (PANCAN) data obtained from UCSC Xena. Gene expression matrices were transformed as log2(TPM + 1) and mapped to Gene Symbols. Immune and stromal cell infiltration scores were calculated using the deconvo_epic function in the IOBR R package. For stomach adenocarcinoma (STAD), the ESTIMATE R package was used to calculate ESTIMATE scores, which reflect tumor purity, stromal content, and immune infiltration based on gene expression data. STAD samples were subsequently divided into high- and low-RRN3-expression groups. Immune cell infiltration in STAD was further assessed using the single-sample Gene Set Enrichment Analysis (ssGSEA) algorithm implemented in the GSVA R package, based on gene signatures for 24 immune cell types. Enrichment scores were calculated for each immune cell type, and differences between the high- and low-RRN3-expression groups were compared to explore immune-related features associated with RRN3 expression.

### Drug response association analysis

The association between *RRN3* expression and drug sensitivity was systematically evaluated using the GSCALite platform, which integrates data from the GDSC and CTRP databases. FDA-approved drugs potentially associated with *RRN3* were identified using DrugBank, and drug–gene interactions were visualized with Cytoscape (version 3.7.2). To identify small-molecule compounds potentially associated with *RRN3*-related transcriptional signatures, differential expression analysis between the high- and low-*RRN3*-expression groups was performed using DESeq2 in R based on TCGA RNA-seq data. The top 100 upregulated and top 100 downregulated genes were then submitted to the Connectivity Map (CMap) database to identify candidate compounds associated with *RRN3*-related transcriptional profiles.

### Construction of the lncRNA–miRNA–*RRN3* regulatory network

Potential miRNAs targeting *RRN3* were predicted by integrating data from the DIANA-microT, miRWalk, and TargetScan databases. Only miRNAs identified by all three databases were retained for subsequent analysis. Interactions between these miRNAs and their potential upstream lncRNAs were further explored using the StarBase v2.0 database, and expression correlations were also evaluated. The analysis was restricted to *Homo sapiens* (genome version hg19), with a minimum of five supporting CLIP-seq datasets, and RNA degradation data were included when available. Sankey diagrams illustrating miRNA–lncRNA interactions were generated using the Xiantao bioinformatics platform. A putative lncRNA–miRNA–*RRN3* regulatory network was then constructed and visualized using Cytoscape.

### Co-expression and functional enrichment analysis of *RRN3*

RNA-seq data from the TCGA cohort were analyzed using the stats package in R to identify genes co-expressed with *RRN3*. Genes with a Pearson correlation coefficient > 0.3 and a P-value < 0.05 were considered significantly correlated and were visualized using heatmaps. The top 100 positively correlated genes were submitted to the STRING database to construct a protein–protein interaction (PPI) network, and core modules and hub genes were identified using MCODE and CytoHubba in Cytoscape. In addition, the top 300 positively correlated genes were subjected to Gene Ontology (GO) and Kyoto Encyclopedia of Genes and Genomes (KEGG) enrichment analyses to explore *RRN3*-related biological processes and pathways.

### Patients and specimens

A total of 30 gastric cancer patients who underwent surgical resection at the First Affiliated Hospital of Fujian Medical University between March 2011 and March 2016 were included in this study. Paired GC tissues and matched adjacent normal tissues were collected from these patients. Among these paired samples, 30 pairs were used for qRT-PCR analysis, six pairs were used for western blotting, and formalin-fixed paraffin-embedded sections from 30 paired GC and adjacent normal tissues were used for immunohistochemical analysis. None of the enrolled patients received preoperative radiotherapy or chemotherapy.

Clinicopathological information, including age, sex, pathological stage, depth of tumor invasion, lymph node metastasis, tumor differentiation, vascular invasion, perineural invasion, Lauren classification, growth pattern, and H. pylori infection status, was collected from electronic medical records and postoperative pathological reports. Lauren classification, growth pattern, and H. pylori infection status were extracted from postoperative pathological reports. The histopathological features were reviewed by two experienced pathologists who were blinded to RRN3 staining results. The use of these archived clinical specimens and clinical data was approved by the Ethics Committee of the First Affiliated Hospital of Fujian Medical University [approval number: 2018(030)]. Written informed consent was obtained from all patients. This study was conducted in accordance with the Declaration of Helsinki.

### Cell culture and lentiviral transduction

The human GC cell lines AGS and MKN45 were obtained from the Chinese Academy of Sciences (Shanghai, China). The normal gastric epithelial cell line GES-1 was used as a non-malignant control. AGS cells were cultured in F-12 medium, whereas GES-1 and MKN45 cells were maintained in RPMI-1640 medium. Both media were supplemented with 10% fetal bovine serum (FBS), penicillin, and streptomycin, and cells were cultured at 37 °C in a humidified incubator with 5% CO_2_.

Two independent shRNA lentiviral vectors targeting regions outside the coding sequence of RRN3 and the corresponding negative control vector (LV-Ctrl) were purchased from GeneChem (Shanghai, China).

The shRNA target sequences were:

sh-RRN3#1, 5′-GGAAGGATTTGCTTGTTTAAT-3′;

sh-RRN3#2, 5′-GGGACTTCAGAGACCAATAAA-3′.

Lentiviral particles were generated by co-transfecting HEK293T cells with the expression vector together with packaging and envelope plasmids. Forty-eight hours after transfection, viral supernatants were collected and used to infect target cells. Stably transduced cells were selected with puromycin to establish *RRN3* knockdown cell lines.

For rescue experiments, RRN3 was re-expressed by transfection with the pCDH-RRN3 plasmid, which contains the RRN3 coding sequence. The pCDH-RRN3 plasmid was also purchased from GeneChem (Shanghai, China). Because the shRNA target sequences were located outside the coding sequence of RRN3, the exogenous RRN3 transcript was not targeted by these shRNAs. RRN3 knockdown and overexpression were verified by western blotting.

### Quantitative real-time PCR

To evaluate RRN3 mRNA expression levels in clinical tissue samples and cell lines, total RNA was extracted using TRIzol reagent (Life Technologies, USA) according to the manufacturer’s instructions. Complementary DNA (cDNA) was synthesized using the Evo M-MLV RT Kit (AG11707, Accurate Biology, China). Quantitative real-time PCR was then performed using the SYBR Green Premix Pro Taq HS qPCR Kit (AG11701, Accurate Biology, China) on a real-time PCR system. GAPDH was used as the internal control, and relative expression levels were calculated using the 2^−ΔΔCt method. The primer sequences were as follows: *RRN3* forward, 5′-ACTGGCTGCTAGAATTCCGTT-3′; reverse, 5′-AACTGTACATGGCAGGAGGC-3′. *GAPDH* forward, 5′-GTCTCCTGCGACTTCAACAGCA-3′; reverse, 5′-ACCACCCTGTTGCTGTAGCCGT-3′.

### Western blotting

Total protein was extracted from clinical tissue samples and cultured cells and quantified using the BCA Protein Assay Kit (P0010, Beyotime, China). Equal amounts of protein (50 μg per lane) were separated by 10% SDS-PAGE. Electrophoresis was performed at 90 V for 30 min and then at 110 V until adequate protein separation was achieved. Proteins were subsequently transferred onto methanol-activated PVDF membranes at 110 V for 1 h at 4 °C. The membranes were blocked with 5% non-fat milk for 1 h and then incubated overnight at 4 °C with primary antibodies against RRN3 (rabbit, 25918-1-AP, 1:1000, Proteintech), Beta Actin (mouse, 66009-1-Ig, 1:20000, Proteintech) and Beta Tubulin (rabbit, 10094-1-AP, 1:2000, Proteintech). After washing three times with TBST (10 min each), the membranes were incubated with HRP-conjugated secondary antibodies (goat anti-rabbit IgG, RGAR001, 1:3000; goat anti-mouse IgG, SA00001-1, 1:2000) for 1 h at room temperature. Protein bands were visualized using an enhanced chemiluminescence (ECL) detection system. Band intensities were quantified using ImageJ software and normalized to Beta Actin or Beta Tubulin.

### Cell proliferation and colony formation assays

Cell proliferation was assessed using the Cell Counting Kit-8 (CCK-8; Dojindo, Japan). Briefly, 1,000 cells per well were seeded into 96-well plates and cultured at 37 °C in a humidified incubator with 5% CO_2_. At the indicated time points (0, 24, 48, 72, and 96h), CCK-8 working solution was added to each well and incubated for 2 h, after which absorbance was measured at 450 nm. For the colony formation assay, 1000 cells were seeded into each well of 6-well plates and cultured under standard conditions for 2 weeks. The colonies were then fixed with methanol, stained with crystal violet, and colonies containing more than 50 cells were counted.

### Wound healing assay

AGS and MKN45 cells from the control, RRN3-knockdown, and RRN3-rescue groups were seeded into 6-well plates at a density of 1.0 × 10^6^ cells per well and cultured at 37 °C in a humidified incubator with 5% CO_2_. When the cells reached approximately 90% confluence, a linear wound was created using a sterile 200 μL pipette tip. After washing with PBS to remove detached cells, the cultures were maintained in serum-free medium. Images of the wound area were captured at 0 and 24 h under a light microscope, and the wound closure rate was calculated accordingly.

### Transwell migration and invasion assays

Transwell migration and invasion assays were performed using AGS and MKN45 cells from the control, RRN3-knockdown, and RRN3-rescue groups. For the migration assay, cells suspended in serum-free medium were seeded into the upper chamber of Transwell inserts, whereas the lower chamber was filled with F-12 or RPMI-1640 medium containing 20% FBS as a chemoattractant. After 48 h of incubation, the cells were fixed with methanol and stained with crystal violet. Non-migrated cells on the upper surface of the membrane were gently removed, and the migrated cells on the lower surface were counted under an inverted microscope. For the invasion assay, the upper chamber was precoated with Matrigel, while the remaining procedures were performed as described for the migration assay.

### Immunohistochemistry

Immunohistochemistry was performed on formalin-fixed paraffin-embedded tissue sections. Briefly, tissue sections were deparaffinized, rehydrated, and subjected to antigen retrieval using citrate buffer. Endogenous peroxidase activity was blocked with hydrogen peroxide, and nonspecific binding was blocked with normal serum. The sections were then incubated overnight at 4 °C with an anti-RRN3 antibody (rabbit, 25918-1-AP, 1:1000, Proteintech), followed by incubation with a secondary antibody. The signal was developed using DAB, and the sections were counterstained with hematoxylin.

RRN3 immunostaining was evaluated according to staining intensity and the percentage of positive tumor cells. Staining intensity was scored as 0, negative; 1, weak; 2, moderate; and 3, strong. The percentage of positive cells was scored as 0, 0%; 1, 1–25%; 2, 26–50%; 3, 51–75%; and 4, 76–100%. The final IHC score was calculated by multiplying the intensity score by the percentage score. The staining results were independently evaluated by two investigators who were blinded to the clinicopathological data.

### Tumor xenografts

All animal experiments were conducted in accordance with the Guidelines for the Care and Use of Laboratory Animals of the NIH. The experiments were performed at the Animal Experiment Center of Fujian Medical University and approved by the Institutional Animal Care and Use Committee of Fujian Medical University (IACUC FJMU 2023-Y0735).

For the subcutaneous xenograft assay, BALB/c nude mice were randomly divided into different groups, with five mice per group. In brief, 5 × 10^6^ stably transfected GC cells with RRN3 overexpression, RRN3 knockdown, or corresponding control treatment were suspended in PBS and subcutaneously injected into the right axillary region of nude mice. Tumor length (L) and width (W) were measured every 3 days using calipers, and tumor volume was calculated using the formula: volume = 0.5 × L × W². At 21 days after tumor implantation, the mice were euthanized, and the tumors were harvested, photographed, weighed, and subjected to further analysis.

### Statistical analysis

Bioinformatic analyses were performed using the above-mentioned online platforms and R software (version 4.2.1). Statistical analyses of the experimental data were conducted using GraphPad Prism 10 (GraphPad Software, La Jolla, CA, USA). Data are presented as the mean ± standard deviation (SD). Differences between two groups were assessed using Student’s t-test. Associations between RRN3 expression and clinicopathological parameters were analyzed using the chi-square test or Fisher’s exact test, as appropriate. A two-sided *P* < 0.05 was considered statistically significant. Statistical significance is indicated as follows: *P < 0.05, **P < 0.01, ***P < 0.001, and ****P < 0.0001.

## Results

### Subcellular localization of RRN3 protein and expression of *RRN3*

**Figure 2:**
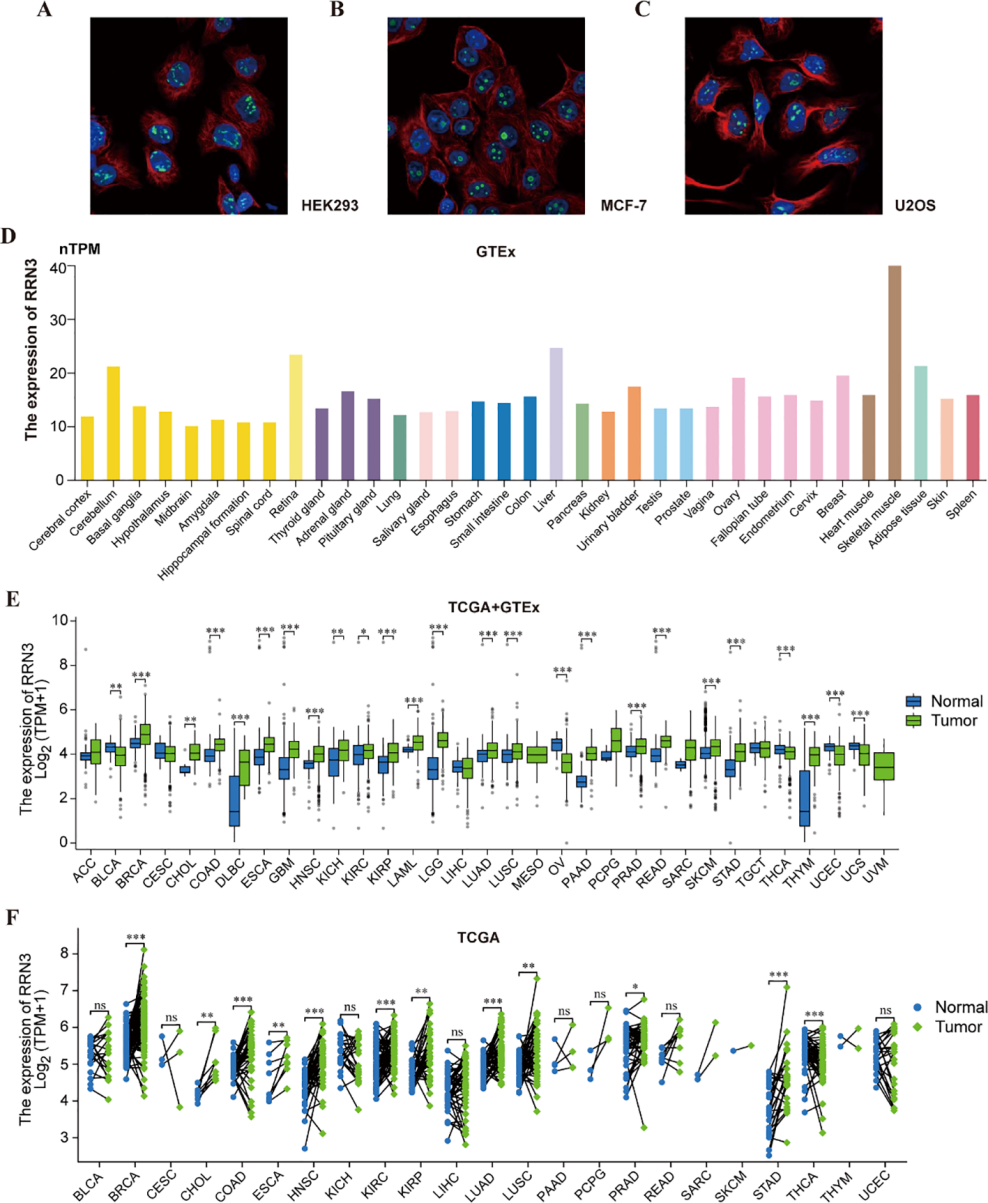
Subcellular localization and expression levels of RRN3. **(A–C)** Immunofluorescence images showing the subcellular localization of RRN3 protein in HEK293 **(A)**, MCF-7 **(B)**, and U2OS **(C)** cells based on data from the HPA database. Images in panels **A–C** were reproduced from the Human Protein Atlas (HPA, proteinatlas.org), available under the Creative Commons Attribution-ShareAlike 4.0 International License (CC BY-SA 4.0). Image credit: Human Protein Atlas. **(D)** Expression levels of RRN3 in normal human tissues based on GTEx data integrated in the HPA database. **(E)** Differential expression of RRN3 between tumor and normal tissues in the combined TCGA–GTEx dataset. **(F)** Comparison of *RRN3* expression between tumor tissues and paired adjacent normal tissues in the TCGA cohort. ns, not significant; *P < 0.05, **P < 0.01, and ***P < 0.001.

Subcellular localization analysis showed that RRN3 was predominantly localized to the perinucleolar region (**Figures 2A–C****).** Data from the HPA database indicated that RRN3 was widely expressed across normal human tissues, with relatively high levels in the retina, liver, and skeletal muscle (**Figure 2D****;** **Supplementary Table 1**). Integrated TCGA and GTEx analyses demonstrated that *RRN3* expression was significantly upregulated (*P* < 0.05) in multiple cancer types, including BRCA, CHOL, COAD, DLBC, ESCA, GBM, KICH, KIRP, LAML, LGG, LUAD, LUSC, PRAD, READ, SKCM, STAD, THYM, PAAD, and HNSC, whereas decreased expression was observed in BLCA, OV, UCS, THCA, and UCEC (**Figure 2E****;** **Supplementary Table 2**). Analysis of paired TCGA samples further confirmed significant differences in *RRN3* expression between tumor tissues and adjacent normal tissues in BRCA, CHOL, COAD, ESCA, HNSC, KIRC, KIRP, LUAD, LUSC, PRAD, STAD, and THCA (**Figure 2F****;** **Supplementary Table 3**). In addition, GEPIA2 analysis revealed a significant association between *RRN3* expression and tumor stage in KIRP and OV (*P* < 0.05; **Supplementary Figure 1**). Collectively, these findings indicate that RRN3 is predominantly localized to the perinucleolar region, is broadly expressed in normal tissues, and is aberrantly expressed in multiple cancer types, including GC.

### Association of RRN3 expression with survival outcomes and ROC curve profiles across cancers

**Figure 3:**
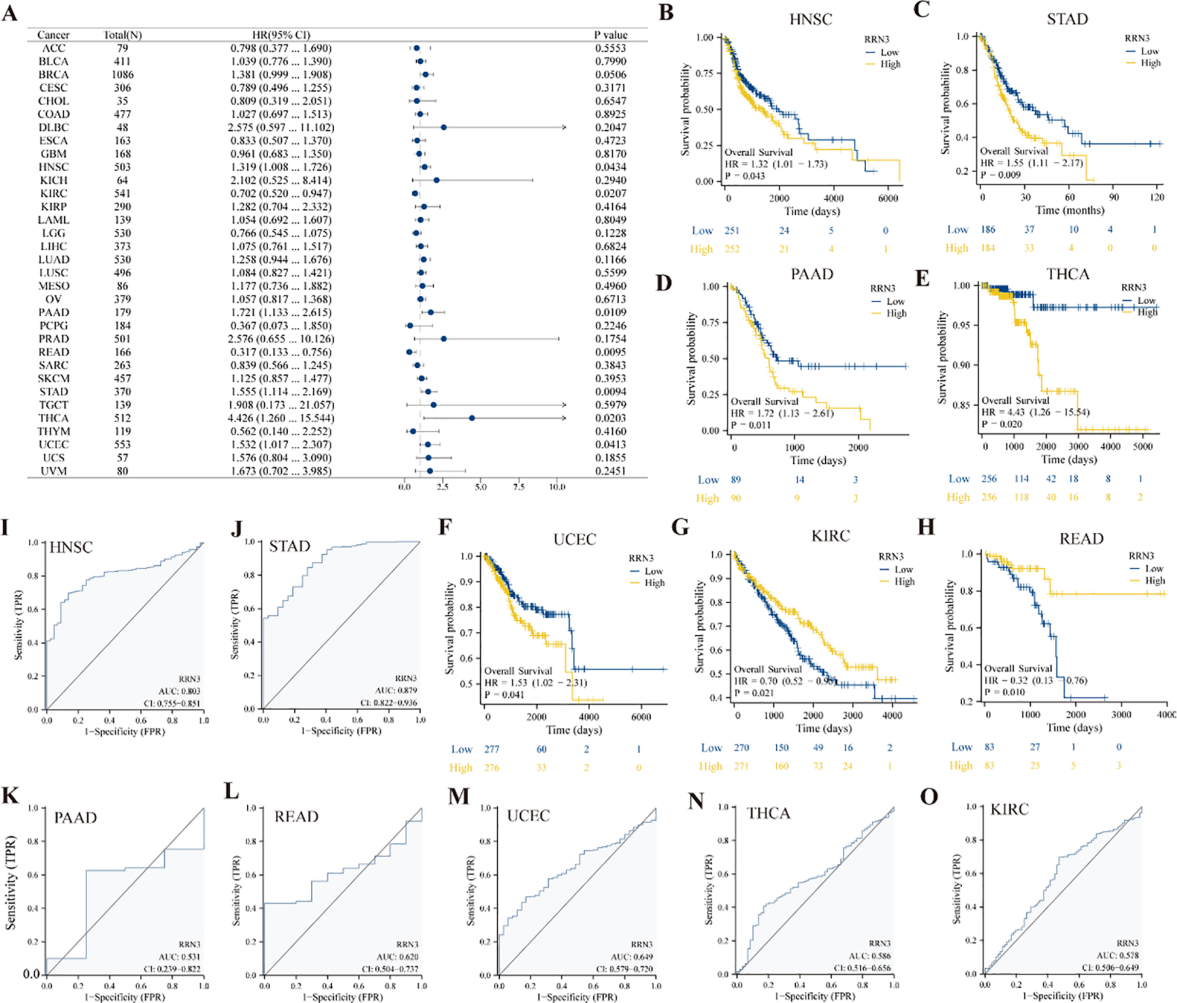
Association of RRN3 expression with survival outcomes and ROC curve profiles across cancers. **(A)** Forest plot showing the association between RRN3 expression and overall survival across multiple cancer types based on Cox regression analysis. **(B–H)** Kaplan–Meier survival curves showing the associations between RRN3 expression and overall survival in HNSC **(B)**, STAD **(C)**, PAAD **(D)**, THCA **(E)**, UCEC **(F)**, KIRC **(G)**, and READ **(H)**. **(I–O)** Receiver operating characteristic (ROC) curves showing the ability of RRN3 expression to distinguish tumor tissues from normal tissues in HNSC **(I)**, STAD **(J)**, PAAD **(K)**, READ **(L)**, UCEC **(M)**, THCA **(N)**, and KIRC **(O)**.

Cox proportional hazards regression analysis showed that high *RRN3* expression was associated with unfavorable survival outcomes in multiple cancer types, with statistically significant associations observed in PAAD and STAD (*P* < 0.05, HR > 1) (**Figure 3A**). In contrast, elevated *RRN3* expression was associated with favorable survival outcomes in KIRC and READ (*P* < 0.05, HR < 1). Kaplan–Meier survival analysis further showed that high *RRN3* expression was associated with unfavorable overall survival in several malignancies, including HNSC (**Figure 3B**; *P* = 0.043), STAD (**Figure 3C**; *P* = 0.009), PAAD (**Figure 3D**; *P* = 0.011), THCA (**Figure 3E**; *P* = 0.020), and UCEC (**Figure 3F**; *P* = 0.041). In contrast, in KIRC (**Figure 3G**; *P* = 0.021) and READ (**Figure 3H**, *P* = 0.010), low *RRN3* expression was associated with poorer overall survival, suggesting that the association between *RRN3* expression and survival outcomes may vary across tumor types. To evaluate the ability of RRN3 expression to distinguish tumor tissues from normal tissues in public datasets, receiver operating characteristic (ROC) curves were constructed for each cancer type. The results showed that *RRN3* had relatively high AUC values in HNSC (AUC = 0.803; **Figure 3I**) and STAD (AUC = 0.879; **Figure 3J**). In PAAD, READ, UCEC, THCA, and KIRC, the AUC values were lower than 0.75, suggesting weaker tumor-normal separation in these cancer types (**Figures 3K–O**). Collectively, these findings indicate that *RRN3* expression is associated with survival outcomes across multiple cancer types and shows tumor-normal separation ability in GC public datasets.

### Genetic alteration analysis of *RRN3*

**Figure 4:**
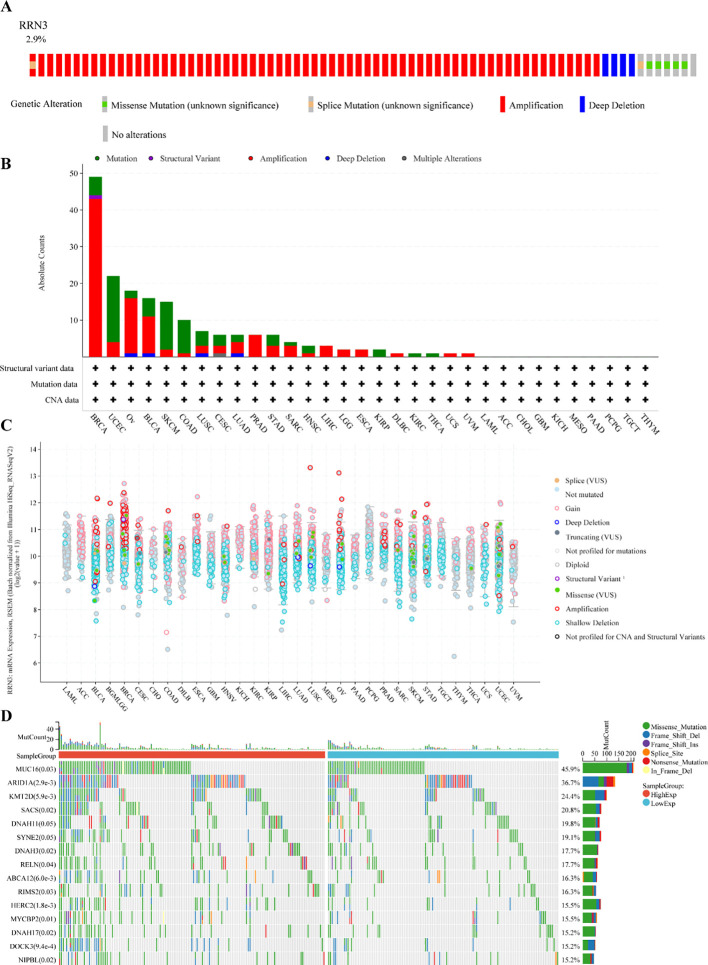
Genetic alteration analysis of RRN3. **(A)** Frequency of RRN3 alterations across pan-cancer, with an overall alteration rate of 2.9%. **(B)** Distribution and types of RRN3 alterations across different cancer types. **(C)** Association between RRN3 mRNA expression and putative copy-number alterations (CNAs) across pan-cancer. **(D)** Top 15 genes with differential mutation frequencies between the high- and low-RRN3-expression groups in STAD.

The genetic alteration landscape of *RRN3* across pan-cancer was analyzed using the cBioPortal platform. As shown in **Figure 4A**, *RRN3* alterations were identified in 79 of 2,565 patients, corresponding to an overall alteration frequency of approximately 3%. These alterations were distributed across 32 cancer types (**Figure 4B**), with the highest alteration frequency observed in BRCA (4.52%). In STAD, the alteration frequency of *RRN3* was 1.36%, representing a moderate level among the analyzed cancer types. Overall, copy number amplification was the predominant form of alteration, followed by point mutations. We further evaluated the association between *RRN3* copy number alterations and *RRN3* mRNA expression (**Figure 4C**), which showed that RRN3 mRNA expression tended to increase with copy number amplification. To investigate the mutational landscape associated with *RRN3* expression in STAD, samples were divided into high- and low-*RRN3*-expression groups (**Figure 4D**). A total of 15 genes showed significantly different mutation frequencies between the two groups. Among them, the five most significantly altered genes were *MUC6*, *ARID1A*, *KMT2D*, *SACS*, and *DNAH11*, suggesting that *RRN3* expression may be associated with distinct mutational features in STAD.

### Epigenetic association analysis of *RRN3*

**Figure 5:**
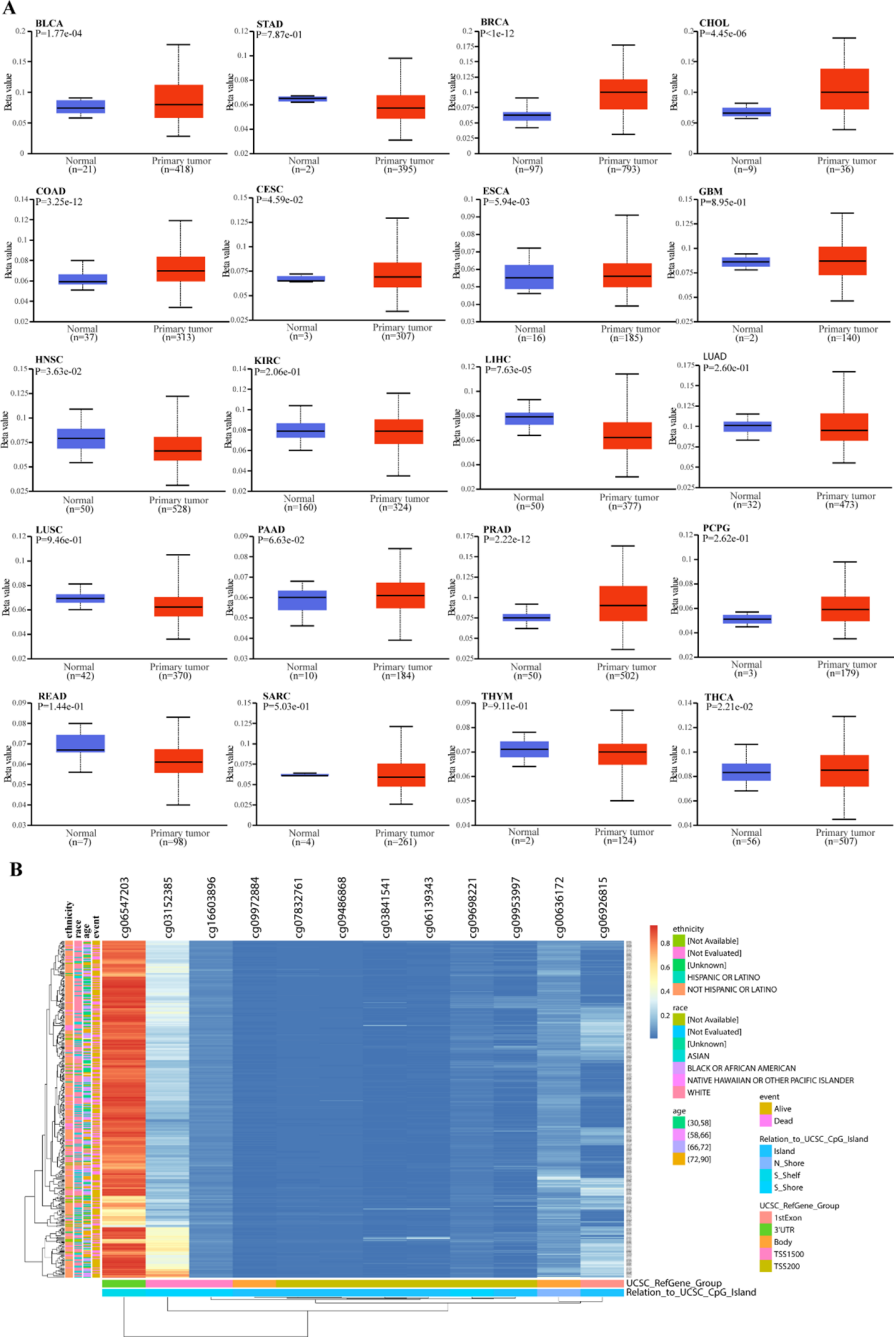
DNA methylation analysis of RRN3. **(A)** Comparison of RRN3 promoter methylation levels between normal and tumor tissues across 20 cancer types based on UALCAN. **(B)** Heatmap showing the methylation levels of RRN3 CpG sites in STAD generated using the MethSurv platform.

DNA methylation levels within the *RRN3* promoter region were evaluated across normal tissues and 20 tumor types. As shown in **Figure 5A**, *RRN3* promoter methylation levels varied across different cancer types. In STAD, the promoter methylation level of RRN3 was slightly lower in tumor tissues than in normal tissues, but this difference was not statistically significant (P = 7.87e-01). Further analysis using the MethSurv platform identified 12 CpG sites within *RRN3* in STAD (**Figure 5B**). Among these, cg06547203 showed a markedly elevated methylation level, whereas the remaining CpG sites generally exhibited relatively low methylation levels.

**Figure 6:**
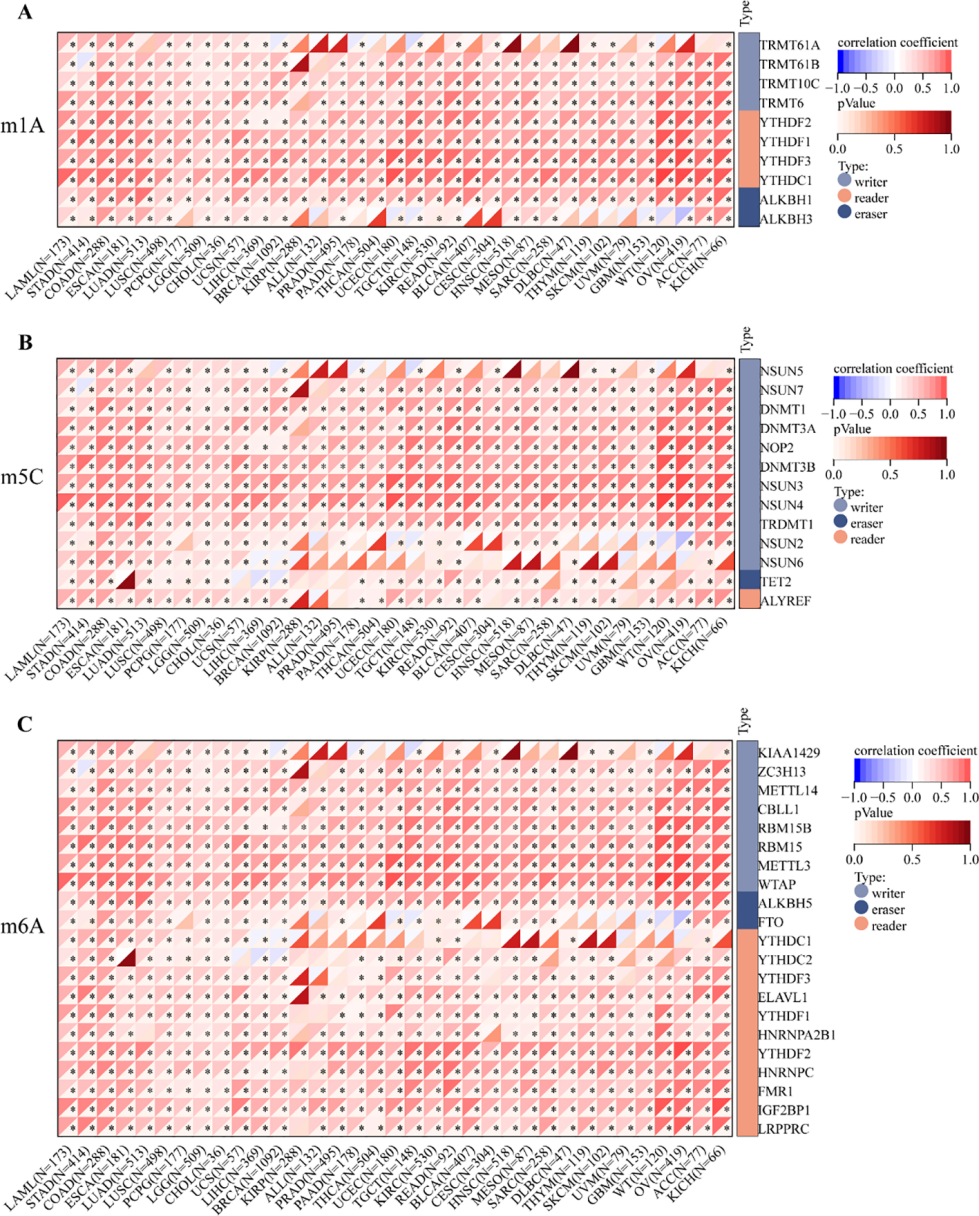
Correlation analysis between *RRN3* expression and RNA methylation regulators. **(A)** Correlation between *RRN3* expression and m1A regulators. **(B)** Correlation between *RRN3* expression and m5C regulators. **(C)** Correlation between *RRN3* expression and m6A regulators. Correlations were evaluated using Pearson’s correlation coefficients, and * statistical significance is indicated as P < 0.05.

To investigate the potential association between *RRN3* expression and RNA methylation regulators, we analyzed 45 genes related to RNA methylation (listed in **Supplementary Table 4**), including writers, erasers, and readers. As shown in **Figures 6A–C**, *RRN3* expression was positively correlated with most m1A-, m5C-, and m6A-related regulators across multiple cancer types. In STAD, *RRN3* expression was significantly and positively correlated with five representative regulators, including CBLL1 (r = 0.684, *P* < 0.001), YTHDF3 (r = 0.652, *P* < 0.001), RBM15 (r = 0.643, *P* < 0.001), YTHDC1 (r = 0.638, *P* < 0.001), and NSUN4 (r = 0.635, *P* < 0.001). Collectively, these results indicate that RRN3 exhibits distinct epigenetic and epitranscriptomic associations across multiple cancer types. In STAD, although the overall promoter methylation difference was not statistically significant, the site-specific CpG methylation pattern and the significant positive correlations between RRN3 expression and RNA methylation regulators suggest that RRN3 may have potential epigenetic relevance in GC.

### Association between *RRN3* expression and immune-related features

**Figure 7:**
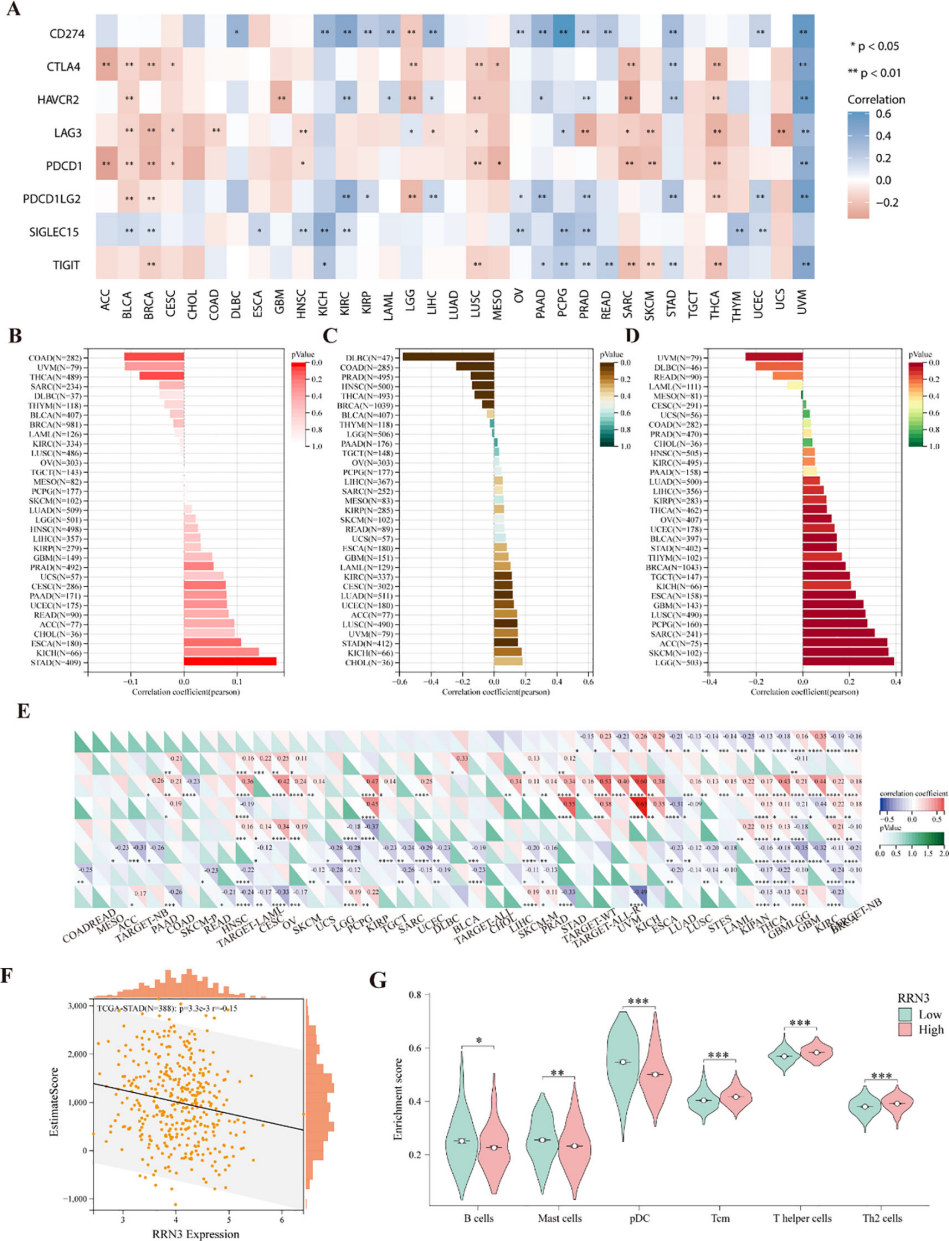
Association between RRN3 expression and immune-related features. **(A)** Correlation between RRN3 expression and immune checkpoint genes across multiple cancer types. **(B–D)** Associations of RRN3 expression with tumor mutation burden (TMB, B), microsatellite instability (MSI, C), and tumor purity **(D)**. **(E)** Correlation between RRN3 expression and immune and stromal cell infiltration. **(F)** Association between RRN3 expression and ESTIMATE scores in STAD. **(G)** Comparison of enrichment scores for different immune cell subsets between the high- and low-RRN3-expression groups in STAD. *P < 0.05; **P < 0.01; ***P < 0.001; ****P < 0.0001.

Immune checkpoint (ICP) genes play important roles in regulating immune cell infiltration and influencing the efficacy of immunotherapy. To investigate the association between *RRN3* expression and immune-related features across multiple cancer types, we first evaluated its correlation with eight ICP genes, including CD274, CTLA4, HAVCR2, LAG3, PDCD1, PDCD1LG2, SIGLEC15, and TIGIT. As shown in **Figure 7A** and **Supplementary Table 5**, *RRN3* expression was positively correlated with these ICP genes in several tumor types, including UVM and STAD, whereas negative correlations were observed in THCA, SARC, and LUSC. In addition, higher *RRN3* expression was significantly associated with higher tumor mutation burden (TMB) in STAD (*P* < 0.05; **Figure 7B**; **Supplementary Table 6**). *RRN3* expression was also positively correlated with microsatellite instability (MSI) in multiple cancers, including STAD, CESC, LUAD, KIRC, and LUSC (*P* < 0.05; **Figure 7C**; **Supplementary Table 7**), whereas negative correlations were observed in COAD, BRCA, PRAD, HNSC, THCA, and DLBC. Regarding tumor purity, *RRN3* expression showed a positive correlation with tumor purity in multiple cancer types, including GBM, LGG, BRCA, ESCA, SARC, STAD, LUSC, THCA, TGCT, PCPG, SKCM, BLCA, and ACC, whereas a significant negative association was observed in UVM (*P* < 0.05; **Figure 7D**; **Supplementary Table 8**).

Further analyses showed that *RRN3* expression was associated with the infiltration of multiple immune and stromal cell types in the tumor microenvironment, with relatively prominent correlations observed in GBM and THCA (**Figure 7E**). In STAD, ESTIMATE analysis revealed a negative correlation between *RRN3* expression and ESTIMATE scores (**Figure 7F**), suggesting a potential association with tumor purity and immune-related features in STAD. Moreover, ssGSEA analysis showed significant differences between the high- and low-*RRN3*-expression groups in STAD for B cells, mast cells, plasmacytoid dendritic cells (pDCs), central memory T cells (Tcm), T helper cells, and Th2 cells (**Figure 7G**). No significant differences were observed for the remaining immune cell subsets.

### Drug sensitivity association analysis

**Figure 8:**
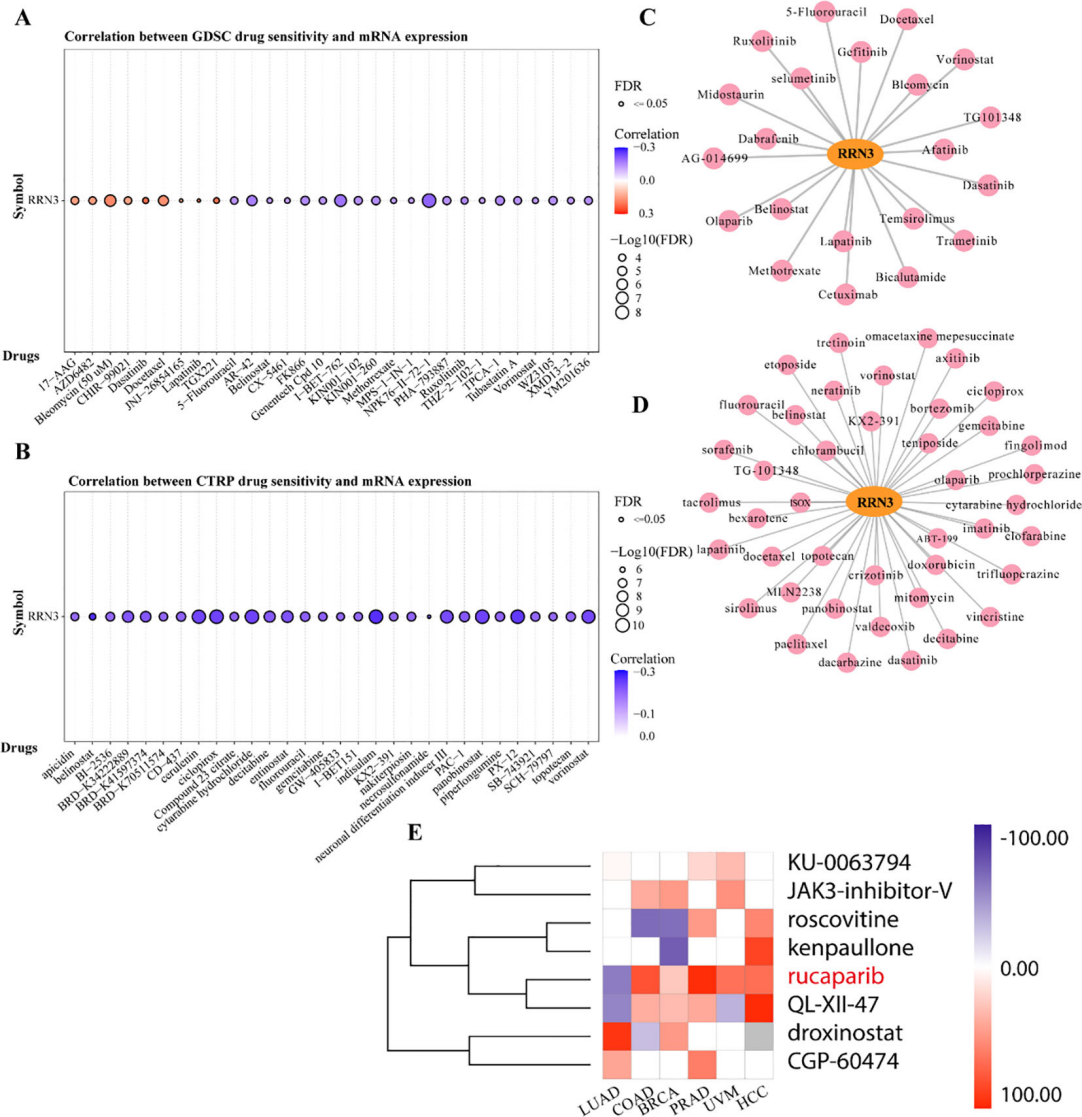
Drug sensitivity association analysis related to RRN3 expression across multiple cancer types. **(A)** Association between RRN3 expression and drug sensitivity based on GDSC data. **(B)** Association between RRN3 expression and drug sensitivity based on CTRP data. **(C)** FDA-approved anticancer drugs potentially associated with RRN3 expression identified from the GDSC dataset. **(D)** FDA-approved anticancer drugs potentially associated with RRN3 expression identified from the CTRP dataset. **(E)** Heatmap showing candidate small-molecule compounds predicted from Connectivity Map (CMap) analysis based on RRN3-related transcriptional signatures.

Drug sensitivity analysis based on the GDSC database showed that *RRN3* expression was associated with cellular responses to multiple anticancer agents. Among these, dasatinib, TGX221, and lapatinib showed the strongest positive correlations with *RRN3* expression (**Figure 8A****;** **Supplementary Table 9**), whereas NPK76-II-72-1, I-BET-762, and AR-42 showed negative correlations. Similar results were observed in the CTRP database, in which *RRN3* expression was positively associated with sensitivity to dasatinib, JW-55, and BRD-K99006945, but negatively associated with GSK-J4, belinostat, and indisulam (**Figure 8B****;** **Supplementary Table 10**). By integrating results from the GDSC and CTRP databases and focusing on compounds with consistent associations, we identified 21 and 42 FDA-approved anticancer drugs potentially associated with *RRN3* expression, respectively (**Figures 8C, D****;** **Supplementary Tables 11**, **12**). Among these, dasatinib showed a particularly strong positive correlation with *RRN3* expression. To further explore candidate small-molecule compounds, we used the “Query” module of the Connectivity Map (CMap) database to analyze differentially expressed genes between the high- and low-*RRN3*-expression groups. This analysis identified 15 classes of small-molecule compounds targeting pathways such as ALK, BCR-ABL, BTK, CDK, and MET (**Supplementary Table 13**). The top eight candidate compounds ranked by connectivity score are shown in **Figure 8E**. These findings suggest that RRN3-associated transcriptional signatures may help identify candidate compounds with potential therapeutic relevance, although further experimental validation is required.

### Prediction of a putative *lncRNA–miRNA*–*RRN3* regulatory network in STAD

**Figure 9:**
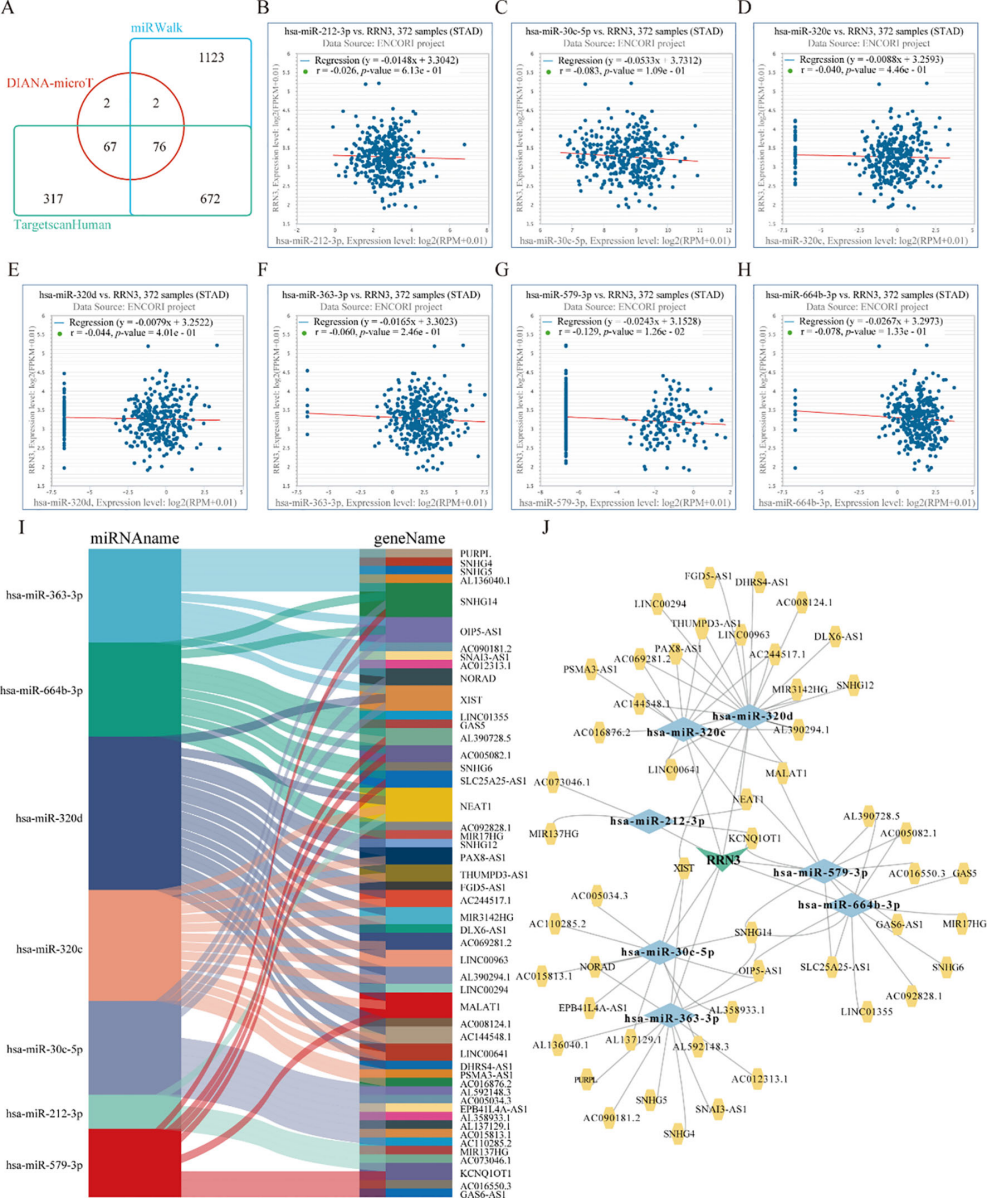
Prediction of a putative *RRN3*-associated ceRNA network in STAD. **(A)** Venn diagram showing the predicted *RRN3*-targeting miRNAs identified by DIANA-microT, miRWalk, and TargetScan. **(B–H)** Scatter plots showing the correlations between *RRN3* expression and the predicted miRNAs, including *hsa-miR-212-3p* **(B)***, hsa-miR-30c-5p* **(C)***, hsa-miR-320c* **(D)***, hsa-miR-320d* **(E)***, hsa-miR-363-3p* **(F)***, hsa-miR-579-3p* **(G)***, and hsa-miR-664b-3p* **(H)**. **(I)** Sankey diagram showing the relationships between the predicted miRNAs and their corresponding lncRNAs. **(J)** lncRNA–miRNA–*RRN3* regulatory network in STAD constructed using Cytoscape.

To identify potential upstream regulators of *RRN3* in STAD, three online prediction databases, DIANA-microT, miRWalk, and TargetScan, were used to predict miRNAs that may target *RRN3* (**Figure 9A****;** **Supplementary Table 14**). A total of 147, 1873, and 1132 candidate miRNAs were obtained from DIANA-microT, miRWalk, and TargetScan, respectively, among which 76 miRNAs overlapped across all three datasets and were considered potential *RRN3*-targeting miRNAs. We next explored the upstream lncRNAs associated with these 76 candidate miRNAs using the StarBase v2.0 database. The results showed that 14 miRNAs, including *hsa-miR-212-3p, hsa-miR-25-3p, hsa-miR-30c-5p, hsa-miR-3167, hsa-miR-320b, hsa-miR-320c, hsa-miR-320d, hsa-miR-323b-3p, hsa-miR-363-3p, hsa-miR-367-3p, hsa-miR-381-3p, hsa-miR-513b-5p, hsa-miR-579-3p*, and *hsa-miR-664b-3p*, were predicted to interact with multiple lncRNAs, suggesting possible upstream regulatory relationships. Because mRNAs generally show inverse expression patterns with their targeting miRNAs, we further evaluated the correlations between *RRN3* expression and these 14 candidate miRNAs. Among them, seven miRNAs, namely *hsa-miR-212-3p* (**Figure 9B**), *hsa-miR-30c-5p* (**Figure 9C**), *hsa-miR-320c* (**Figure 9D**)*, hsa-miR-320d* (**Figure 9E**)*, hsa-miR-363-3p* (**Figure 9F**)*, hsa-miR-579-3p* (**Figure 9G**), and *hsa-miR-664b-3p* (**Figure 9H**), showed significant negative correlations with *RRN3* expression, suggesting that they may act as candidate upstream regulators of *RRN3*. In addition, lncRNAs may function as competitive endogenous RNAs (ceRNAs) that indirectly regulate mRNA expression through miRNA sequestration. We therefore identified lncRNAs predicted to interact with these seven miRNAs and observed inverse associations between the lncRNAs and their corresponding miRNAs (**Figure 9I**). Based on these findings, a putative lncRNA–miRNA–*RRN3* regulatory network was generated (**Figure 9J**), providing preliminary clues to the post-transcriptional regulation of RRN3 in STAD.

### Functional analysis of *RRN3* co-expressed genes in STAD

**Figure 10:**
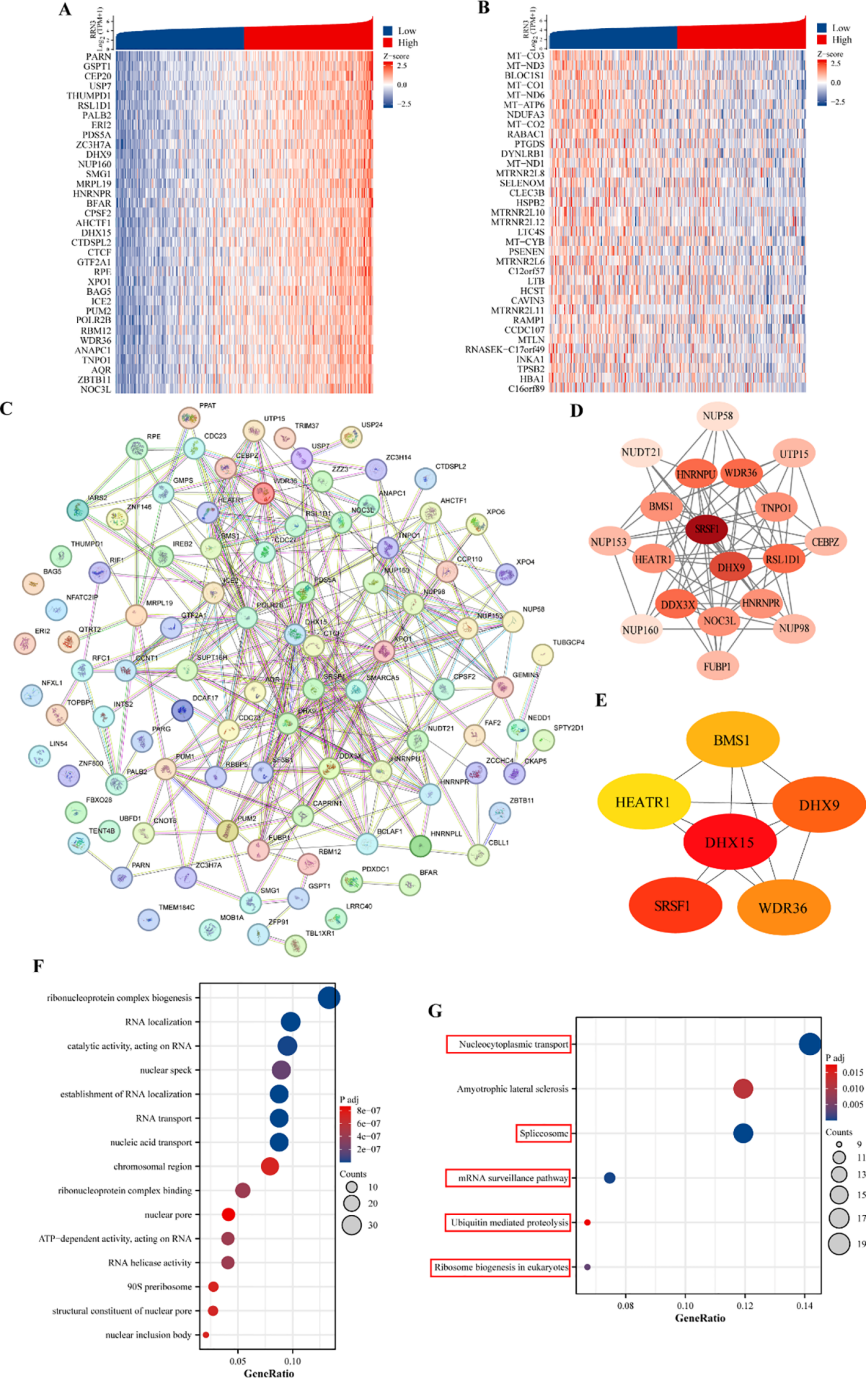
Co-expressed genes and functional analysis of *RRN3* in STAD. **(A, B)** Heatmaps showing the top 35 positively **(A)** and negatively **(B)** correlated genes associated with *RRN3* expression in STAD. **(C)** Protein–protein interaction (PPI) network constructed using STRING based on the top 100 positively correlated genes. **(D)** Core module identified by MCODE analysis of the PPI network. **(E)** Top six hub genes identified by CytoHubba analysis. **(F, G)** Gene Ontology (GO, F) and Kyoto Encyclopedia of Genes and Genomes (KEGG, G) enrichment analyses of the top 300 positively co-expressed genes associated with *RRN3*.

To investigate the potential biological functions of *RRN3* in STAD, we analyzed genes co-expressed with *RRN3* using TCGA data. Heatmaps were generated to display the top 35 positively and negatively correlated genes associated with *RRN3* expression in STAD (**Figures 10A, B****;** **Supplementary Table 15**). A protein–protein interaction (PPI) network was then constructed using the STRING database based on the top 100 positively correlated genes (**Figure 10C**). Using the MCODE plugin in Cytoscape, a core module containing 19 hub genes was identified, including *SRSF1, DHX9, RSL1D1, WDR36, HNRNPU, DDX3X, TNPO1, BMS1, HEATR1, NOC3L, HNRNPR, FUBP1, NUP98, CEBPZ, UTP15, NUP153, NUP58, NUDT21*, and *NUP160* (**Figure 10D**). Further analysis using the CytoHubba plugin identified six top-ranking hub genes, namely *DHX15, SRSF1, DHX9, WDR36, BMS1*, and *HEATR1* (**Figure 10E**). GO enrichment analysis of the top 300 positively co-expressed genes revealed significant enrichment in biological process (BP), cellular component (CC), and molecular function (MF) categories (**Figure 10F**; **Table 1**). KEGG pathway analysis showed that these genes were mainly enriched in nucleocytoplasmic transport, spliceosome, mRNA surveillance pathway, ubiquitin-mediated proteolysis, and ribosome biogenesis in eukaryotes (**Figure 10G**; **Table 2**). Collectively, these results suggest that *RRN3* may be associated with multiple biological processes and pathways related to GC progression.

**Table 1:** Gene Ontology (GO) analyses of the top 300 co-expression genes positively associated with RRN3 expression.

Ontology	ID	Description	Gene ratio	Bg ratio	P value	P adjust
BP	GO:0006403	RNA localization	28/285	196/18800	2.08E-19	4.87135E-16
BP	GO:0050657	nucleic acid transport	25/285	159/18800	1.7814E-18	1.39068E-15
BP	GO:0050658	RNA transport	25/285	159/18800	1.7814E-18	1.39068E-15
BP	GO:0051236	establishment of RNA localization	25/285	162/18800	2.83852E-18	1.66196E-15
BP	GO:0022613	ribonucleoprotein complex biogenesis	38/285	448/18800	5.89264E-18	2.76011E-15
CC	GO:0016607	nuclear speck	26/290	411/19594	5.46517E-10	1.87455E-07
CC	GO:0098687	chromosomal region	23/290	366/19594	6.39397E-09	7.34029E-07
CC	GO:0030686	90S preribosome	8/290	29/19594	6.85268E-09	7.34029E-07
CC	GO:0042405	nuclear inclusion body	6/290	12/19594	8.5601E-09	7.34029E-07
CC	GO:0005643	nuclear pore	12/290	93/19594	1.26157E-08	8.6544E-07
MF	GO:0140098	catalytic activity, acting on RNA	28/294	388/18410	3.02676E-11	1.18649E-08
MF	GO:0043021	ribonucleoprotein complex binding	16/294	150/18410	2.38913E-09	4.18741E-07
MF	GO:0003724	RNA helicase activity	12/294	77/18410	3.20465E-09	4.18741E-07
MF	GO:0008186	ATP-dependent activity, acting on RNA	12/294	79/18410	4.3405E-09	4.25369E-07
MF	GO:0017056	structural constituent of nuclear pore	8/294	28/18410	9.05951E-09	7.10265E-07

**Table 2:** Kyoto Encyclopedia of Genes and Genome enrichment (KEGG) analyses of the top 300 co-expression genes positively associated with RRN3 expression.

Ontology	ID	Description	Gene ratio	Bg ratio	P value	P adjust
KEGG	hsa03013	Nucleocytoplasmic transport	19/134	108/8164	6.48288E-15	1.30E-12
KEGG	hsa03040	Spliceosome	16/134	147/8164	1.77604E-09	1.78492E-07
KEGG	hsa03015	mRNA surveillance pathway	10/134	97/8164	3.79184E-06	0.000254053
KEGG	hsa03008	Ribosome biogenesis in eukaryotes	9/134	109/8164	7.03483E-05	0.003535001
KEGG	hsa05014	Amyotrophic lateral sclerosis	16/134	364/8164	0.000289497	0.011637791
KEGG	hsa04120	Ubiquitin mediated proteolysis	9/134	142/8164	0.000522528	0.01750468

### Clinical validation and functional role of RRN3 in GC

**Figure 11:**
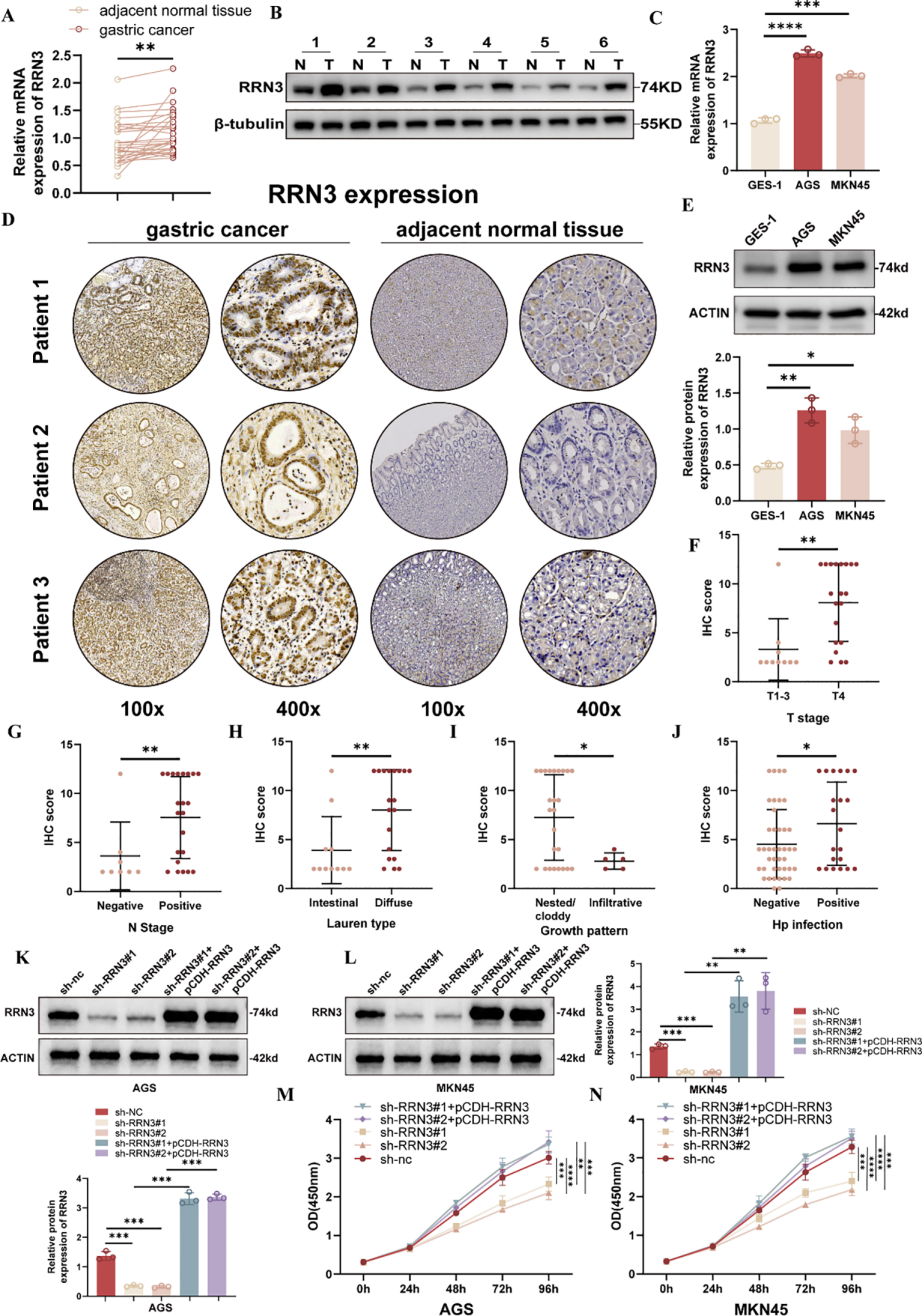
Clinical validation of RRN3 expression and effects of RRN3 knockdown and re-expression on GC cell proliferation. **(A)** RRN3 mRNA expression in paired GC tissues and adjacent normal tissues detected by qRT-PCR (n = 30 pairs). **(B)** RRN3 protein expression in paired GC tissues and adjacent normal tissues detected by western blotting (n = 6 pairs). **(C)** RRN3 mRNA expression in GES-1, AGS, and MKN45 cells. **(D)** Representative IHC images showing RRN3 expression in GC tissues and adjacent normal tissues (n = 30 pairs). **(E)** RRN3 protein expression in GES-1, AGS, and MKN45 cells detected by western blotting. **(F–J)** Associations between RRN3 IHC scores and clinicopathological features, including T stage **(F)**, N stage **(G)**, Lauren classification **(H)**, growth pattern **(I)**, and H. pylori infection status **(J)** (n = 30). **(K, L)** Western blotting showing RRN3 knockdown and re-expression in AGS **(K)** and MKN45 **(L)** cells. **(M, N)** CCK-8 assays showing the effects of RRN3 knockdown and re-expression on the proliferation of AGS **(M)** and MKN45 **(N)** cells. *P < 0.05; **P < 0.01; ***P < 0.001; ****P < 0.0001.

To further validate RRN3 expression in GC, we examined RRN3 levels in clinical tissue samples and GC cell lines. qRT-PCR analysis showed that RRN3 mRNA expression was significantly higher in GC tissues than in paired adjacent normal tissues (**Figure 11A**). Western blotting using six paired tissue samples also showed increased RRN3 protein expression in GC tissues compared with adjacent normal tissues (**Figure 11B**). Consistently, RRN3 expression was upregulated in AGS and MKN45 cells compared with the normal gastric epithelial cell line GES-1 at both the mRNA and protein levels (**Figures 11C, E**). Immunohistochemical staining further showed higher RRN3 expression in GC tissues than in adjacent normal tissues (**Figure 11D**). Clinicopathological analysis showed that higher RRN3 IHC scores were associated with advanced T stage, positive lymph node metastasis, Lauren classification, growth pattern, and H. pylori infection status (**Figures 11F–J**). These results suggest that RRN3 is upregulated in GC and may be associated with GC progression in this clinical cohort.

**Figure 12:**
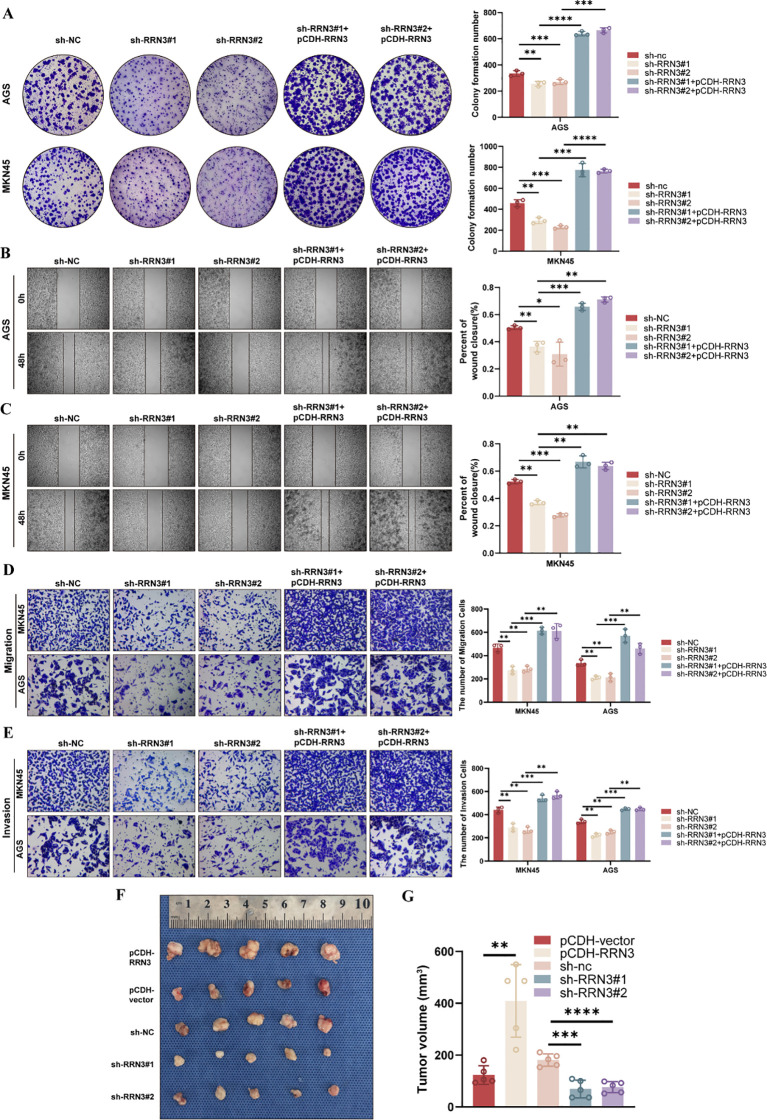
RRN3 promotes malignant phenotypes of GC cells *in vitro* and tumor growth *in vivo*. **(A)** Colony formation assays showing the effects of RRN3 knockdown and re-expression on the clonogenic ability of AGS and MKN45 cells. **(B, C)** Wound healing assays showing the effects of RRN3 knockdown and re-expression on the migration of AGS **(B)** and MKN45 **(C)** cells. **(D, E)** Transwell assays showing the effects of RRN3 knockdown and re-expression on the migration **(D)** and invasion **(E)** of AGS and MKN45 cells. **(F)** Representative images of xenograft tumors from the pCDH-RRN3, pCDH-vector, sh-NC, sh-RRN3#1, and sh-RRN3#2 groups. **(G)** Quantification of xenograft tumor volumes in different groups. *P < 0.05; **P < 0.01; ***P < 0.001; ****P < 0.0001.

To investigate the functional role of RRN3 in GC cells, we established stable RRN3-knockdown AGS and MKN45 cells using two independent shRNAs. To further verify the specificity of the knockdown effects, rescue experiments were performed by re-expressing RRN3 using the pCDH-RRN3 plasmid. Western blotting confirmed efficient RRN3 knockdown in the sh-RRN3#1 and sh-RRN3#2 groups and restored RRN3 expression in the rescue groups in both AGS and MKN45 cells (**Figures 11K, L**). CCK-8 assays showed that RRN3 knockdown significantly inhibited the proliferation of AGS and MKN45 cells, whereas RRN3 re-expression reversed this inhibitory effect (**Figures 11M, N**). Colony formation assays further showed that RRN3 knockdown reduced the clonogenic ability of AGS and MKN45 cells, while RRN3 re-expression restored colony formation (**Figure 12A**). Wound healing assays showed that RRN3 knockdown suppressed cell migration, and this effect was reversed by RRN3 re-expression in both AGS and MKN45 cells (**Figures 12B, C**). Consistently, Transwell assays demonstrated that RRN3 knockdown reduced the migratory and invasive abilities of GC cells, whereas RRN3 re-expression rescued these phenotypes (**Figures 12D, E**). These findings indicate that RRN3 promotes the proliferation, migration, and invasion of GC cells.

To further evaluate the role of RRN3 in tumor growth *in vivo*, a subcutaneous xenograft model was established using GC cells with RRN3 overexpression, RRN3 knockdown, or corresponding control treatment. Compared with the pCDH-vector group, the pCDH-RRN3 group formed larger tumors. In contrast, tumors in the sh-RRN3#1 and sh-RRN3#2 groups were smaller than those in the sh-NC group (**Figure 12F**). Quantitative analysis showed that RRN3 overexpression significantly increased tumor volume, whereas RRN3 knockdown significantly reduced tumor volume (**Figure 12G**). These results suggest that RRN3 contributes to GC tumor growth *in vivo*.

## Discussion

In the present study, we systematically investigated the expression pattern, clinical relevance, and biological function of *RRN3* in GC and across multiple cancer types. Pan-cancer analysis showed that *RRN3* was aberrantly expressed in several tumor types and was significantly upregulated in GC. This finding was further supported by clinical tissue validation, including qRT-PCR, western blotting, and immunohistochemistry. Higher *RRN3* expression was also associated with several clinicopathological features related to GC progression. In addition, RRN3 expression showed prognostic associations, epigenetic and epitranscriptomic associations, immune-related features, drug sensitivity-related signatures, and a putative ceRNA regulatory network. Functional experiments further demonstrated that RRN3 knockdown inhibited GC cell proliferation, migration, and invasion, whereas RRN3 re-expression partly rescued these effects. The *in vivo* xenograft assay also supported a role for RRN3 in promoting tumor growth. Collectively, these findings suggest that RRN3 is associated with GC progression and may represent a candidate molecule for further investigation in GC.

We found that *RRN3* was upregulated in many cancer types, including GC, compared with normal tissues. This finding is consistent with recent studies showing that RRN3 overexpression is associated with malignant characteristics and poor prognosis in pancreatic cancer, and with the broader concept that enhanced ribosome biogenesis and Pol I transcriptional activity contribute to tumor progression. Interestingly, *RRN3* expression was relatively low in several tumor types, including BLCA, OV, THCA, UCEC, and UCS, indicating that the contribution of *RRN3* to tumorigenesis may be tissue-dependent. At present, direct evidence linking low *RRN3* expression to the biological behavior of these cancers remains limited, and the underlying mechanisms warrant further investigation. Notably, ROC curve analysis showed relatively high AUC values for distinguishing GC tissues from normal tissues in public datasets. Its elevated expression was also associated with unfavorable overall survival in public datasets. In addition, our clinical tissue validation further showed that RRN3 was upregulated in GC tissues and was associated with several clinicopathological features related to tumor progression. These observations suggest that RRN3 may have clinical relevance in GC, mainly as a molecule associated with GC progression. However, given the limited size of the clinical cohort and the lack of complete follow-up data, the diagnostic and prognostic value of RRN3 should be interpreted cautiously and requires validation in larger independent cohorts.

Genetic alterations may influence gene expression and protein function during tumor development. In this study, RRN3 showed a relatively low alteration frequency across cancer types, with the highest frequency observed in BRCA (4.52%), suggesting that genomic alterations may not be the major cause of RRN3 dysregulation. This finding is consistent with previous reports (20). In STAD, stratified analysis based on RRN3 expression identified several genes with differential mutation frequencies between the high- and low-RRN3-expression groups. Among them, *MUC6* and *ARID1A* were two representative altered genes. *ARID1A*, a key component of the SWI/SNF chromatin-remodeling complex, is frequently mutated in multiple cancers (23–26). *MUC6* has also been implicated in tumorigenesis. Liu et al. reported that *MUC6* acts as a tumor suppressor by promoting autophagy-dependent degradation of β-catenin, whereas disruption of this process facilitates tumor progression (27). The higher mutation frequencies of *MUC6* and *ARID1A* in the high-*RRN3*-expression group suggest that elevated *RRN3* expression may be associated with specific mutational backgrounds in STAD. However, whether these mutations directly influence RRN3 expression or function requires further investigation.

Epigenetic and epitranscriptomic modifications, including DNA methylation and RNA methylation, can regulate gene expression at transcriptional and post-transcriptional levels without altering the DNA sequence itself (28–30). In the present study, we evaluated the methylation status of the *RRN3* promoter across 20 cancer types and corresponding normal tissues. In GC, no significant difference was observed in the overall promoter methylation level of RRN3 between tumor and normal tissues. This result indicates that global promoter methylation changes are unlikely to fully explain the upregulation of RRN3 in GC. However, site-specific CpG methylation analysis showed heterogeneous methylation patterns within the RRN3 promoter region, suggesting that individual CpG sites may still deserve further investigation. We also assessed the association between *RRN3* expression and regulators of m1A, m^5^C, and m^6^A RNA methylation. *RRN3* expression was positively correlated with the majority of these regulators in multiple cancers, including GC. These findings suggest that RRN3 expression may be associated with epigenetic and epitranscriptomic features during tumor progression. However, these analyses are based on database-derived correlations, and further experimental studies are needed to determine whether DNA methylation or RNA methylation regulators directly contribute to RRN3 dysregulation in GC.

Immunotherapy has become an important therapeutic strategy for advanced GC, but the response to immune checkpoint inhibitors varies considerably among patients because of tumor heterogeneity, molecular features, and differences in the immune microenvironment. At present, PD-L1 expression, MSI/MMR status, TMB, EBV status, and other molecular features have been investigated as biomarkers for immunotherapy response in GC. Among them, PD-L1 expression assessed by immunohistochemistry, especially using the combined positive score, is widely used in clinical practice, while MSI-H/dMMR tumors are generally associated with higher immunogenicity and better responses to immune checkpoint blockade. TMB has also been explored as a potential predictor of immunotherapy response, although its predictive value may vary across patient populations and treatment settings (31–33). In this study, *RRN3* expression in STAD was positively correlated with several immune checkpoint molecules, including *PDCD1* (PD-1) and *CD274* (PD-L1). Upregulation of immune checkpoints is involved in tumor immune evasion (34–36), suggesting that high RRN3 expression may be associated with immune checkpoint-related features in STAD. We further found that *RRN3* expression was positively associated with TMB and MSI. Elevated TMB and MSI are often linked to increased neoantigen load and enhanced tumor immunogenicity (37–40), raising the possibility that tumors with high *RRN3* expression may have distinct immune-related molecular characteristics. However, enhanced immunogenicity does not necessarily translate into effective antitumor immune activation (41). Moreover, PD-L1, TMB, and MSI are not fully interchangeable biomarkers, and a single immune-related marker is often insufficient to accurately predict immunotherapy response in GC (31, 33). Taken together, our data suggest that high RRN3 expression in STAD is associated with immune-related features involving immune checkpoint genes, TMB, MSI, and immune cell infiltration. Direct evidence showing that RRN3 regulates the tumor immune microenvironment is still lacking. Further studies using clinical immunotherapy cohorts, multiplex immunohistochemistry, single-cell sequencing, and mechanistic experiments are needed to clarify whether RRN3 directly contributes to immune regulation and whether it has value in predicting immunotherapy response.

We further explored the relationship between *RRN3* expression and drug sensitivity. Analyses of the GDSC and CTRP databases identified multiple FDA-approved anticancer agents associated with *RRN3* expression. Several of these compounds, including docetaxel, 5-fluorouracil, and mitomycin, have been used in GC treatment. These results suggest that *RRN3* expression may be associated with chemotherapy-related drug-response profiles. Of note, in yeast, RRN3 interacts with the pseudouridine synthase Cbf5p and modulates sensitivity to 5-fluorouracil, suggesting a possible link between RRN3-related rRNA transcription and 5-fluorouracil response (42). Whether a similar mechanism operates in human cancers remains to be determined. Among the identified compounds, dasatinib showed the strongest positive association with *RRN3* expression. Dasatinib is a multi-target tyrosine kinase inhibitor approved for chronic myeloid leukemia and Philadelphia chromosome-positive acute lymphoblastic leukemia, and it also shows anticancer potential in several solid tumors (43–45). Because dasatinib inhibits oncogenic signaling pathways involving SRC family kinases (46), BCR-ABL (47), and c-KIT (48), this observation raises the possibility that dasatinib-related pathways may be linked to RRN3-associated transcriptional features. Moreover, CMap analysis identified several candidate small molecules, among which rucaparib showed the highest enrichment score. As a PARP inhibitor approved for BRCA-mutant tumors (49), rucaparib may be considered a candidate compound for further experimental testing in the context of RRN3-high tumors. Nevertheless, these findings are based on database-derived associations and computational prediction. Further *in vitro*, *in vivo*, and clinical validation is required before RRN3 can be considered a predictive biomarker for drug response in GC.

ceRNA networks have attracted increasing attention in GC because of their role in post-transcriptional regulation. LncRNAs, together with other RNA species such as mRNAs and circular RNAs, may function as “miRNA sponges” by competitively binding miRNAs and thereby reducing their inhibitory effects on target mRNAs. In this study, we identified seven candidate miRNAs potentially interacting with *RRN3* and further screened 50 candidate lncRNAs predicted to bind these miRNAs. Several of these lncRNAs, including *SNHG4 (*50), *SNHG5 (*51), and *SNHG15 (*52), have previously been reported as oncogenic regulators in GC. For example, *SNHG4* promotes GC cell proliferation, migration, invasion, and epithelial–mesenchymal transition through *miR-204-5p (*53); the *SNHG5*/*miR-32* axis modulates GC cell growth and migration through *KLF4* regulation (54); and *SNHG15* contributes to immune evasion in GC via the *miR-141*/*PD-L1* pathway (55). These findings raise the possibility that RRN3 may be embedded in multiple lncRNA–miRNA regulatory axes related to GC progression. Although our ceRNA network is based on bioinformatic prediction and correlation analysis rather than direct experimental validation, it provides a useful framework for future mechanistic studies of *RRN3* regulation in GC.

*RRN3* is a key transcription initiation factor for RNA polymerase I that promotes the recruitment of polymerase I to rDNA promoters and initiates 45S rRNA synthesis, thereby functioning as a central regulator of ribosome biogenesis. Increased ribosome production and protein synthesis are hallmarks of cancer (56, 57), indicating that *RRN3* expression and activity may be closely linked to tumor progression. Previous studies have shown that *RRN3* is regulated by multiple oncogenic signaling pathways and is closely associated with its phosphorylation status (21). When *RRN3* function is disrupted or RNA polymerase I activity is inhibited, cells can undergo nucleolar stress, leading to reduced rRNA synthesis, release of ribosomal proteins, activation of p53 signaling, cell-cycle arrest, and apoptosis (58, 59). This response is particularly pronounced in cancer cells with high biosynthetic demands, suggesting that the RRN3/Pol I axis may represent a biologically relevant vulnerability in cancer. Supporting this concept, a recent study identified NSH76 as a small molecule that directly binds RRN3 and selectively inhibits Pol I transcription by disrupting Pol I pre-initiation complex assembly at the rDNA promoter. NSH76 preferentially suppressed Pol I transcription and proliferation in cancer cells with high RRN3 expression, induced nucleolar stress, and showed limited effects on non-cancerous cells (60). A previous study reported that several natural product-derived small molecules, including sulforaphane, ursolic acid, and betulinic acid, induce nucleocytoplasmic translocation of RRN3, suppress rRNA synthesis, trigger nucleolar stress, and inhibit tumor cell proliferation (61). In the present study, we identified 300 genes co-expressed with *RRN3* in TCGA-STAD samples. GO and KEGG enrichment analyses showed significant enrichment in pathways such as ribosome biogenesis, spliceosome, and mRNA surveillance, suggesting that *RRN3* may occupy a central position in regulatory networks linking transcription, RNA processing, and protein synthesis in GC. Importantly, several hub genes co-expressed with *RRN3*, including *HEATR1*, *SRSF1*, and *DHX9*, have previously been implicated in GC progression (62–64). In line with these bioinformatic findings, our clinical tissue analysis showed that RRN3 was upregulated in GC tissues at both the mRNA and protein levels and that higher RRN3 expression was associated with several clinicopathological features related to tumor progression. Functional experiments further demonstrated that RRN3 knockdown inhibited GC cell proliferation, colony formation, migration, and invasion, whereas RRN3 re-expression partly reversed these inhibitory effects. Moreover, *in vivo* xenograft experiments showed that RRN3 overexpression promoted tumor growth, while RRN3 knockdown reduced tumor growth. These findings provide additional clinical, *in vitro*, and *in vivo* evidence supporting the involvement of RRN3 in GC progression. However, the precise molecular mechanisms by which RRN3 promotes GC progression, whether RRN3 has noncanonical RNA-processing functions in GC, and whether RRN3 can be therapeutically targeted in GC require further investigation.

## Conclusions

In summary, our study shows that RRN3 is aberrantly expressed across multiple cancer types and is significantly upregulated in GC. RRN3 expression was associated with survival outcomes, epigenetic and epitranscriptomic features, immune-related characteristics, drug-response profiles, and a putative ceRNA regulatory network. Clinical tissue analysis showed that RRN3 was upregulated in GC tissues and was associated with several clinicopathological features related to tumor progression. Functional experiments showed that RRN3 knockdown inhibited GC cell proliferation, colony formation, migration, and invasion, whereas RRN3 re-expression partly reversed these effects. *In vivo* xenograft experiments further supported the role of RRN3 in promoting tumor growth. Collectively, these findings suggest that RRN3 is associated with GC progression and may represent a candidate molecule for further investigation in GC.

## Data Availability

Publicly available datasets were analyzed in this study. These data can be found in the databases cited throughout the article, including TCGA, GTEx, HPA, GEPIA2, cBioPortal, UALCAN, MethSurv, GSCALite, DrugBank, and CMap. The experimental data generated in this study are included in the article and its **Supplementary Material**. Further inquiries can be directed to the corresponding authors.

## Supplementary material

Supplementary Figure 1 Association between RRN3 expression and tumor stage across different cancer types. Violin plots show the distribution of RRN3 expression across different tumor stages in multiple cancer types based on GEPIA2 analysis.
