# Supplementary figures and images for: Clinical significance and functional characterization of RRN3 in gastric cancer: insights from pan-cancer analysis and experimental validation
Source: Front Oncol. 2026 May 22;16:1832473. doi: 10.3389/fonc.2026.1832473 (PMC13213868; doi:10.3389/fonc.2026.1832473)

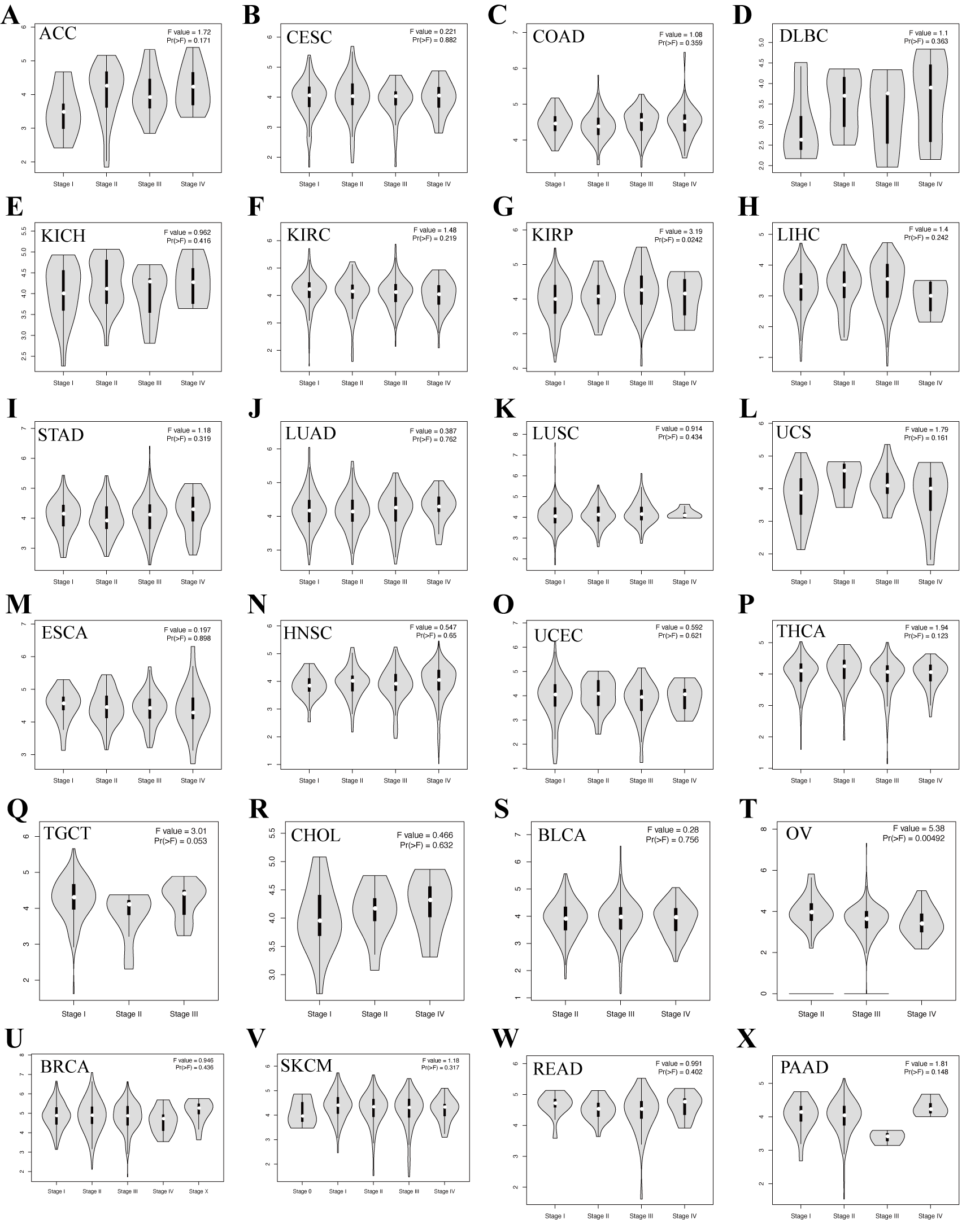

Supplement: Supplementary Figure 1 — Association between RRN3 expression and tumor stage across different cancer types. Violin plots show the distribution of RRN3 expression across different tumor stages in multiple cancer types based on GEPIA2 analysis. [file Image1.tif]
